# ALKBH5-mediated m^6^A modification of lincRNA *LINC02551* enhances the stability of DDX24 to promote hepatocellular carcinoma growth and metastasis

**DOI:** 10.1038/s41419-022-05386-4

**Published:** 2022-11-05

**Authors:** Hongwei Zhang, Yachong Liu, Wei Wang, Furong Liu, Weijian Wang, Chen Su, He Zhu, Zhibin Liao, Bixiang Zhang, Xiaoping Chen

**Affiliations:** 1 Hepatic Surgery Center, Tongji Hospital, Tongji Medical College, Huazhong University of Science and Technology, Wuhan, 430030 China; 2 Hubei Key Laboratory of Hepato-Pancreato-Biliary Diseases, Wuhan, 430030 China; 3 Key Laboratory of Organ Transplantation, Ministry of Education and Ministry of Health, Wuhan, 430030 China

**Keywords:** Liver cancer, Oncogenes

## Abstract

As the most important RNA epigenetic regulation in eukaryotic cells, N6-metheyladenosine (m^6^A) modification has been demonstrated to play significant roles in cancer progression. However, this modification in long intergenic non-coding RNAs (lincRNAs) and the corresponding functions remain elusive. Here, we showed a lincRNA *LINC02551* was downregulated by AlkB Homolog 5 (ALKBH5) overexpression in a m^6^A-dependent manner in hepatocellular carcinoma (HCC). Functionally, *LINC02551* was required for the growth and metastasis of HCC. Mechanistically, *LINC02551*, a bona fide m^6^A target of ALKBH5, acted as a molecular adaptor that blocked the combination between DDX24 and a E3 ligase TRIM27 to decrease the ubiquitination and subsequent degradation of DDX24, ultimately facilitating HCC growth and metastasis. Thus, ALKBH5-mediated *LINC02551* m^6^A methylation was required for HCC growth and metastasis.

## Introduction

Hepatocellular carcinoma (HCC) is one of the most common cancer and the third leading cause of cancer-related deaths worldwide [1]. The high mortality rate results from late presentation at an advanced stage, a high incidence of tumor metastasis, and tumor recurrence after surgical resection [2]. Although significant progress has been achieved, very few approaches can be utilized in the clinic to prevent the recurrence and metastasis of HCC [3]. Hence, identifying the molecular mechanisms of HCC pathogenesis and metastasis is of great importance.

LncRNAs are emerging as fundamental to cell biology because of their ability to reprogram gene expression and influence distinct cellular functions [4, 5]. And it has been shown that some lncRNAs serve as oncogenes or tumor suppressors [6, 7]. Mechanistically, lncRNAs are decoys for messenger RNAs and microRNAs (miRNAs) [8, 9]. Additionally, although named “non-coding RNAs”, some lncRNAs can encode small peptides [10, 11]. Because of their relatively large size enables them to form complex structures, lncRNAs can interact with proteins and regulate protein-protein interactions [12].

Reversible RNA modification leads to a new level of post-transcriptional regulation of gene expression that is involved in many physiological and pathological processes [13, 14]. N6-methyladenine (m^6^A) is a dynamic and reversible chemical modification and is the most abundant modification of mRNA [15, 16]. m6A is extensively involved in mRNA metabolism, affecting mRNA degradation and stability or translation [17, 18]. AlkB Homolog 5 (ALKBH5), an important “erasers”, plays significant roles in the regulation of gene expression [19, 20]. It has been proved that ALKBH5 specifically demethylates m^6^A-modified RNA and suppresses malignancy in HCC via m^6^A-guided epigenetic inhibition of LYPD1 [21]. m^6^A RNA deposition is initiated by “writers”, and the modification is recognized by “readers”, both of which are conserved and preferentially bind an RR (m^6^A) CU (R = G or A) consensus motif [22]. A kind of “readers” belong to the insulin-like growth factor (IGF) family, and they specially bind m^6^A-modified RNA and regulate RNA stability [23, 24]. However, the mechanisms through which m^6^A regulates lncRNA activity in HCC need to be further explored [25–27].

In this study we identified *LINC02551* as the downstream target of ALKBH5, which regulated its expression in an m^6^A-dependent manner. Insulin-like growth factor 2 mRNA binding protein 1 (IGF2BP1) has been implicated in prolonging the *LINC02551* lifespan. *LINC02551* that is located in the nucleus can interact with DDX24 to decrease the TRIM27-induced ubiquitination-induced degradation of DDX24. We found that *LINC02551* promoted HCC progression by stabilizing DDX24 expression. Overall, our study reveals that *LINC02551* is a promising biomarker for prognostic prediction and a potential therapeutic target in HCC.

## Materials and methods

### Patients and specimens

Human liver tumor tissues and corresponding adjacent normal tissues were obtained from patients undergoing hepatectomy between 2012 and 2015 at Tongji Hospital Liver Surgery Center, Tongji Medical College, Huazhong University of Science and Technology (HUST; Wuhan, China). Written informed consent was obtained from all patients and this study was approved by the Medical Ethics Committee of Tongji Hospital. All procedures conformed to the standards of the Declaration of Helsinki.

### RNA sequencing

HLF cells were transfected with pcDNA3.1-ALKBH5 or pcDNA3.1-Vector plasmids. After 48 h, total RNA was extracted and transcriptome sequencing was performed by by GeneRead Biotechnology (Wuhan, China) to identify the differentially expressed lncRNAs. The cutoff value of differentially expressed lncRNAs was set as |log2[fold change]| > 1 and *P* < 0.05.

### Plasmids and constructions

To generate lentivirus-based stable overexpression or knockdown cell lines, CDS of ALKBH5 was cloned into the BamHI/SalI sites of pLenti-CMV-Puro plasmid (Addgene #17448) and the short hairpin RNAs (shRNAs) targeting ALKBH5 and *LINC02551* were cloned into the pLKO.1-TRC vector (Addgene #10879).

For the luciferase reporter assay, Full-length of *LINC02551* (wt) or m^6^A mutant (m1, m2, m3, m4) *LINC02551* sequences were cloned into psiCHECK^TM-2^ Vector (Promega, USA). pcDNA3.1 vector was used for the construction of *LINC02551*, ALKBH5, ALKBH5-H204A, IGF2BP1, IGF2BP1-mut, DDX24 and TRIM27 plasmids. siRNAs targeting IGF2BP1 and DDX24 was synthesized by Ribo Biotech (Guangzhou, China). All sequences were verified by DNA Sanger sequencing. The sequences of shRNA and siRNA were listed as follows: sh*LINC02551*-1: 5′- CCGGTGTCAACTCCACCTTTAGACTCGAGTCTAAAGGTGGAGTTGACATTTTTG-3′; sh*LINC02551*-2: 5′-CCGGGTTCAACGGAAATTCACAACTCGAGTTGTGAATTTCCGTTGAACTTTTTG-3′; sh*LINC02551*-3: 5′-CCGGTGCCTTGAATAAAGACGTACTCGAGTACGTCTTTATTCAAGGCATTTTTG-3′; shDDX24-1: 5′-CCGGCGCTCAAGAAAGATGAGGATACTCGAGTATCCTCATCTTTCTTGAGCGTTTTTG-3′; shDDX24-2: 5′-CCGGCCCACGTACCTCGGAGATTTACTCGAGTAAATCTCCGAGGTACGTGGGTTTTTG-3′; shDDX24-3: 5′-CCGGTTTCTGTTCTCTGGCTATTTGCTCGAGCAAATAGCCAGAGAACAGAAATTTTTG-3′.

More detailed methods were described in Supplementary Materials.

## Results

### ALKBH5 downregulates *LINC02551* in an m^6^A-dependent manner

**Fig. 1:**
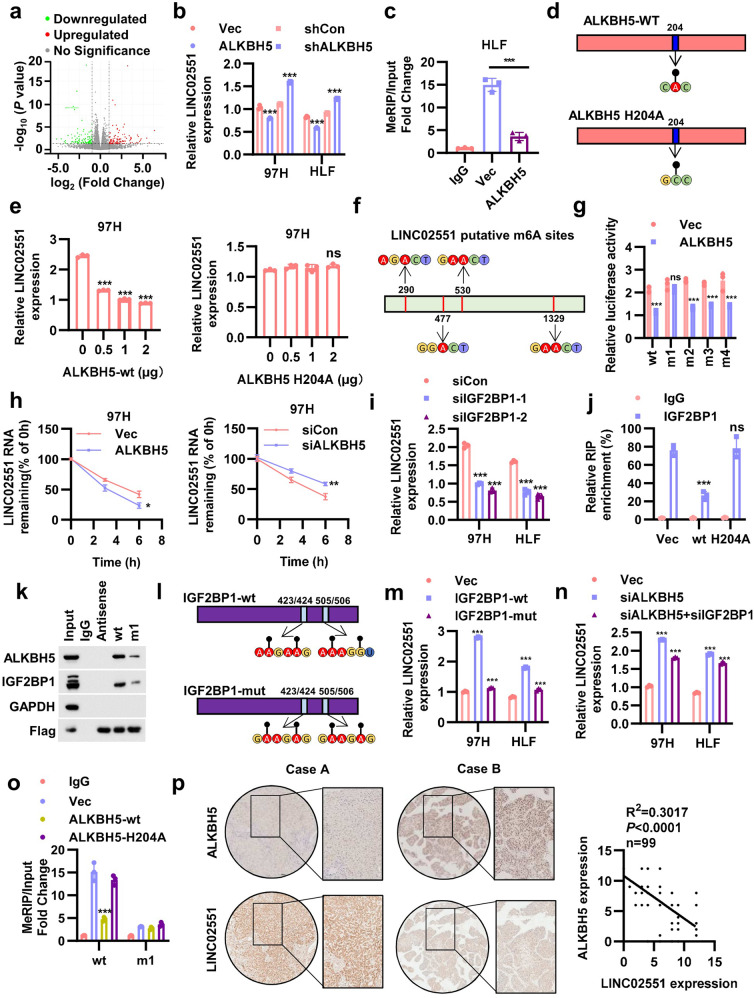
*LINC02551* is regulated by ALKBH5 in an m^6^A -dependent manner. **a** Volcano plot of the analysis of the RNA-seq of HLF cells with and without ALKBH5 overexpression. Red dots indicate the upregulated genes; green dots indicate the downregulated genes. **b** qRT-PCR analysis of *LINC02551* expression in 97H and HLF cells with knockdown and overexpression of ALKBH5. **c** MeRIP results showing *LINC02551* in HLF cells with ALKBH5 overexpression. **d** Schematic diagram showing ALKBH5-wt and ALKBH5-mut. **e** qRT-PCR analysis of *LINC02551* expression in 97H cells transfected with ALKBH5-wt (left panel) and ALKBH5-mut (right panel) via dose gradient. **f** Schematic diagram of *LINC02551* putative m^6^A modification sites. **g** Relative luciferase activity in HLF cells cotransfected with psiCHECK2 containing the *LINC02551* sequence (wt and mutated m^6^A sites) and an ALKBH5 plasmid. **h** The degradation rate of *LINC02551* in 97H cells with ALKBH5 overexpressed (right panel) or knocked down (right panel) and treated with actinomycin D over time. **i** Relative *LINC02551* level in 97H and HLF cells transfected with siIGF2BP1. **j** Abundance of IGF2BP1 targeting *LINC02551* in HLF cells transfected with ALKBH5-wt or ALKBH5-mut. **k** The MS2 RNA pull-down results of *LINC02551*-wt and *LINC02551*-m1. GAPDH was the negative control and Flag was the positive control. **l** Schematic diagram showing IGF2BP1-wt and IGF2BP1-mut. **m** Relative *LINC02551* level in 97H and HLF cells transfected with IGF2BP1-wt or IGF2BP1-mut. **n** Relative *LINC02551* level in 97H and HLF cells transfected with siALKBH5 alone or together with siIGF2BP1. **o** MeRIP results showing *LINC02551*-wt or *LINC02551*-mut in HLF cells with ALKBH5-wt or ALKBH5-mut overexpression. **p** Representative IHC/ISH staining results in paraffin-embedded HCC samples from Tongji Hospital (left panel). The significance of *LINC02551* and ALKBH5 expression is indicated in the right panel. And this analysis was performed using F-test. The remaining statistical analysis was performed using Student’s *t*-tests. Data are mean ± SEM. *P < 0.05, **P < 0.001, ***P < 0.0001.

It has been reported that downregulation of ALKBH5 was associated with poor prognosis in HCC. ALKBH5-mediated m^6^A demethylation led to the posttranscriptional inhibition of LY6/PLAUR Domain Containing 1 (LYPD1), which induced oncogenic behaviors in HCC. To explore how ALKBH5-mediated m^6^A-modified lncRNAs regulation of HCC progression, we applied RNA sequencing (RNA-seq) to HLF cells overexpressing ALKBH5 (Fig. 1a and Fig. S1a). Ten lncRNAs up- or downregulated by ALKBH5 overexpression in 97H and HLF cells were then verified via qRT-PCR (Fig. S1b, c). *LINC02551* appeared to be the top hit because of its concurrent regulation by ALKBH5 in two these HCC cell lines. In 97H and HLF cells, *LINC02551* was negatively regulated by ALKBH5 (Fig. 1b). An Me-RIP assay showed that the extent of m^6^A modification in *LINC02551* was much lower in the ALKBH5 overexpression group (Fig. 1c). Since ALKBH5 is a vital “eraser” in the m^6^A modification process, an inactive ALKBH5-mut (mutant) was utilized to test whether ALKBH5-mediated *LINC02551* regulation is m^6^A dependent [28] (Fig. 1d). When wild-type (ALKBH5-wt) or ALKBH5-mut was overexpressed in dose gradients, *LINC02551* levels were downregulated only in the ALKBH5-wt group, not in the ALKBH5-mut group (Fig. 1e and Fig. S1d, e). According to SRAMP (http://www.cuilab.cn/sramp) analysis, four potential m^6^A sites were identified (with very high confidence) in *LINC02551* (Fig. 1f). To confirm that these four sites are critical for *LINC02551* regulation, point mutations were introduced into putative m^6^A sites: m1, m2, m3, or m4. Then, we constructed a psiCHECK2 plasmid containing *LINC02551*-wt or point mutants. The dual luciferase reporter assay suggested that ALKBH5 did not bind to *LINC02551* when the m1 putative m^6^A site was mutated (Fig. 1g). These results indicate that ALKBH5 might regulate *LINC02551* levels in an m^6^A-dependent manner.

Previous studies have suggested that m^6^A modification mediates different RNA fates. We wondered how the m^6^A modification affected *LINC02551* level. After using Actinomycin D to block RNA transcription, the degradation rate of *LINC02551* decreased when ALKBH5 was knocked down and increased with overexpression of ALKBH5, suggesting that ALKBH5 regulates the decay of *LINC02551* (Fig. 1h). Considering that “readers” directly and critically affect m^6^A-modified transcripts, we investigated potential effectors participating in the process. It has been reported that IGF2BP1 enhanced RNA stability [24]. When IGF2BP1 was knocked down, the *LINC02551* level was decreased (Fig. 1i). RIP assays showed that less IGF2BP1was bound to *LINC02551* in the ALKBH5-wt group but not in the ALKBH5-mut group, which was verified through RNA pull-down assays (Fig. 1j, k). Next, an inactive IGF2BP1-mut was used to verify that IGF2BP1-mediated *LINC02551* regulation is m^6^A-dependent [24] (Fig. 1l). The transfection of IGF2BP1-mut did not lead to the upregulation of *LINC02551* (Fig. 1m). The knockdown of IGF2BP1 partially attenuated the upregulation caused by the knockdown of ALKBH5 (Fig. 1n). Me-RIP assays indicated that the m^6^A modification was enriched to a greater extent on *LINC02551* in the ALKBH5-mut and *LINC02551*-wt groups (Fig. 1o). An IHC analysis showed that the expression of ALKBH5 in HCC patient samples was negatively correlated with the expression of *LINC02551* level (Fig. 1p).

These data suggested that ALKBH5 downregulates *LINC02551* in an m^6^A-dependent manner, and that this effect is mediated by IGF2BP1 recognition.

### *LINC02551* promotes HCC progression in vitro

**Fig. 2:**
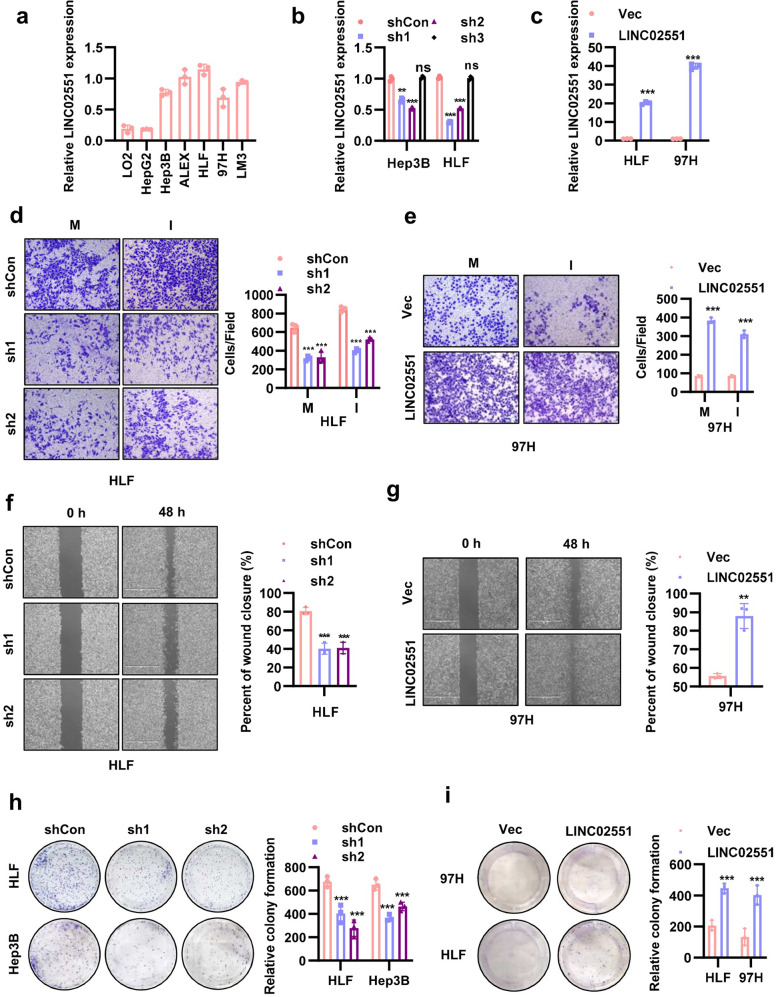
*LINC02551* promotes HCC progression in vitro. **a** Relative *LINC02551* expression determined by qRT-PCR in HCC cell lines. **b** Relative *LINC02551* expression in Hep3B and HLF cells transfected with shRNA-induced *LINC02551* knockdown. **c** Relative *LINC02551* expression in HLF and 97H cells overexpressing *LINC02551*. **d** The migration and invasion results of HLF cells with *LINC02551* knocked down (M: migration; I: invasion). **e** The migration and invasion results with 97H cells overexpressing *LINC02551*. **f** The wound healing assay with HLF cells in which *LINC02551* was knocked down. **g** The wound healing assay with 97H cells overexpressing *LINC02551*. **h** Clonality formation results for the HLF and Hep3B cells with *LINC02551* knocked down. **i** Clonality formation results for the 97H and HLF cells overexpressing *LINC02551*. Statistical analysis was performed using Student’s *t*-tests. Data are mean ± SEM. *P < 0.05, **P < 0.001, ***P < 0.0001.

To explore the function of *LINC02551* in HCC, we checked its expression in different HCC cell lines (Fig. 2a) and constructed stable knockdown cell lines (Fig. 2b) and overexpression cell lines (Fig. 2c). In Hep3B and HLF cells, shRNA-mediated knockdown of *LINC02551* led to decreased cell migration and invasion (Fig. 2d and Fig. S2a). On the contrary, overexpression of *LINC02551* in HLF and 97H cells increased cell motility (Fig. 2e and Fig. S2b). To further verify the effect of *LINC02551* in cell metastasis, the cell wound healing assay was performed and the result indicated that knockdown of *LINC02551* led to decreased cell motility in HLF and Hep3B cells (Fig. 2f and Fig. S2c) and overexpression of *LINC02551* led to increased cell motility (Fig. 2g and Fig. S2d). To determine the effects of *LINC02551* on the cell proliferation, we conducted plate clonality assay and a CCK-8 (Cell Counting Kit 8) assay. The results showed that knockdown of *LINC02551* led to decreased cell colony formation (Fig. 2h) and slower cell growth (Fig. S2e); *LINC02551* overexpression promoted the cell colony formation (Fig. 2i) and led to increased cell proliferation rate (Fig. S2f)

### *LINC02551* accelerates HCC progression in vivo

**Fig. 3:**
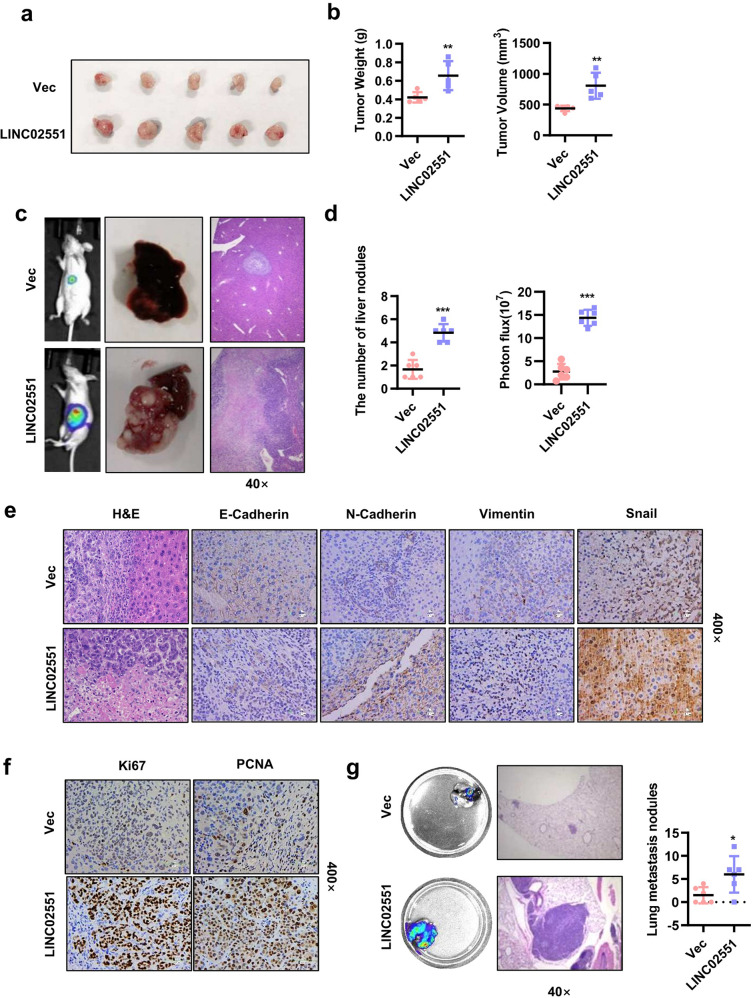
*LINC02551* accelerates HCC progression in vivo. **a** Subcutaneous transplantation model generated with stable 97H-Vec and 97H-*LINC02551* cell lines. When the mice were sacrificed, the tumors were removed. **b** The statistical analysis of the weight and volume of the subcutaneous tumors. **c** The results from the orthotopic transplantation model established with nude mice using 97H-luc-*LINC02551* or vector cells. **d** Statistical analysis of the liver nodule numbers and luciferase activity. **e**, **f** H&E and other stains applied to proteins in liver-bearing tumors. **g** Results of tail vein injection of HLF-luc-*LINC02551* or vector cells in nude mice. After sacrifice, the lungs were removed and photographed. The statistical analysis is presented in the right panel. Statistical analysis was performed using Student’s *t*-tests. Data are mean ± SEM. *P < 0.05, **P < 0.001, ***P < 0.0001.

To evaluate the tumor-promoting role of *LINC02551* in HCC in vivo, we established various xenograft models. Fist, we subcutaneously injected 97H cells overexpressing *LINC02551* in nude mice. Four weeks post-injection, we found that *LINC02551* overexpressing 97H cells caused larger (volume) and heavier (weight) tumor (Fig. 3a, b). We also injected 97H cells overexpressing *LINC02551* in the left lobe of each mouse liver. Five weeks post-inoculation, we examined the intrahepatic metastasis of the 97H cells. Injection of *LINC02551* overexpressing cells substantially increased the capacity of the HCC cells to form secondary lesions in the liver (Fig. 3c, d). IHC staining showed higher N-cadherin, Vimentin, Snail, Ki67, and PCNA signals and weaker E-cadherin signals in the livers of the *LINC02551* overexpression group (Fig. 3e, f). In the lung metastasis model, control or *LINC02551* overexpressing cells were injected into the tail vein of the mice. Six weeks after injection, more lesions were observed in the mice injected with the *LINC02551* overexpressing cells than in those injected with the control cells (Fig. 3g).

These results suggested that *LINC02551* promoted the progression of HCC in vivo.

### *LINC02551* interacts with DDX24 and reduces its ubiquitination degradation

**Fig. 4:**
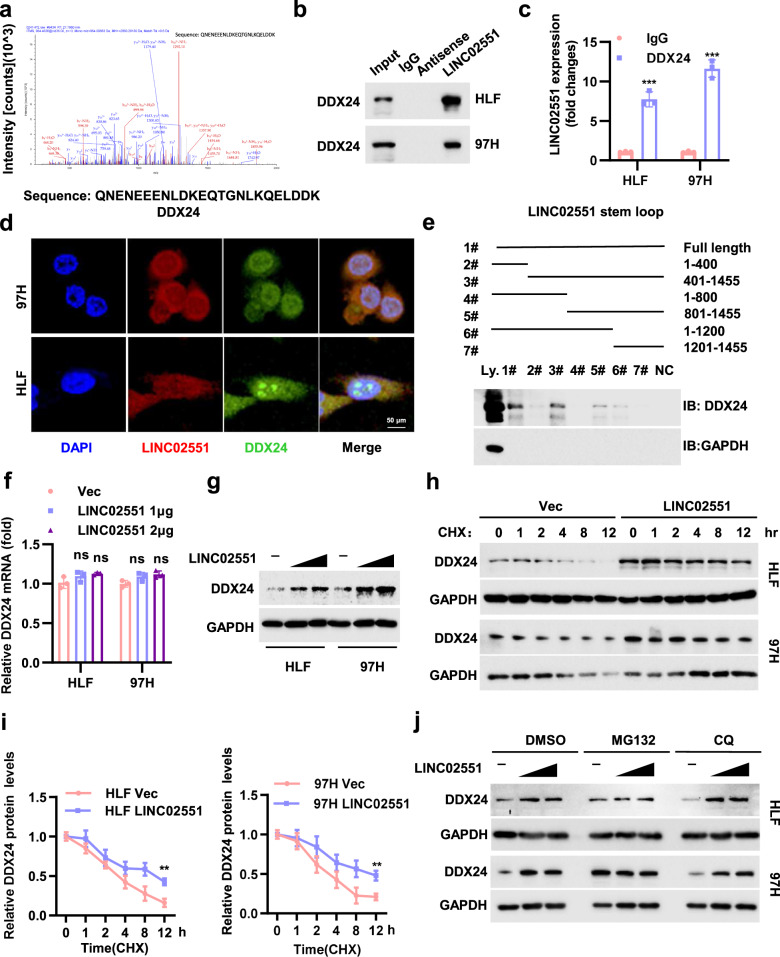
*LINC02551* decreased the ubiquitination-induced degradation of DDX24. **a** Representative peptide sequences of DDX24. **b** The RNA pull-down assay of the combination between *LINC02551* and DDX24. **c** The RIP results of the interaction between DDX24 and *LINC02551*. **d** IF assay coupled with FISH was performed. **e** The RNA pull-down assay showing the combination between truncation *LINC02551* mutants and DDX24. **f**, **g** Relative DDX24 RNA **(f)** and protein levels **(g)** in HLF and 97H cells transfected with *LINC02551* in dose gradients. **h**, **i** WB and statistical analysis of DDX24 expression in HLF and 97H cells after CHX (cycloheximide, 40 μg/ml) treatment. **j** HLF and 97H cells treated with DMSO, proteasome inhibitor MG132 (20 μM) or the lysosome inhibitor CQ (Chloroquine, 100 μM) for 4 h. The endogenous DDX24 level was detected by WB. Statistical analysis was performed using Student’s *t*-tests. Data are mean ± SEM. *P < 0.05, **P < 0.001, ***P < 0.0001, ns means no significance.

We next explored the underlying mechanism of *LINC02551*-induced promotion of HCC progression. RNA pull-down assays followed by mass spectrometry (MS) led to the identification of potential *LINC02551* interaction partners (Table S1). DDX24, a member of the DEAD box-containing RNA helicases family, was verified through analysis (Fig. 4a). RIP assays coupled with RNA pull-down assays were performed to confirm the interaction between *LINC02551* and DDX24 (Fig. 4b, c). Immunostaining showed that *LINC02551* was associated with DDX24 in the nucleus (Fig. 4d). Further, a pull-down analysis showed that DDX24 interacted with an 800-1200 bp sequence of *LINC02551* (Fig. 4e). *LINC02551* overexpression caused an increase in the protein level of DDX24 but not its RNA level (Fig. 4f, g). Therefore, we speculated that *LINC02551* increased DDX24 expression at the posttranscriptional level, probably by attenuating DDX24 degradation. Cycloheximide (CHX) chase results verified our hypothesis (Fig. 4h, i). The protein degradation process involves at least three main systems: the ubiquitin-proteasome, autophagosome and lysosome pathways. The *LINC02551*-induced increase in DDX24 was blocked by MG132 (a proteasome inhibitor) treatment but not by CQ (a lysosome inhibitor) (Fig. 4j). These data suggested that *LINC02551* might attenuate the ubiquitination-mediated degradation of DDX24.

### *LINC02551* attenuates TRIM27-induced DDX24 degradation

**Fig. 5:**
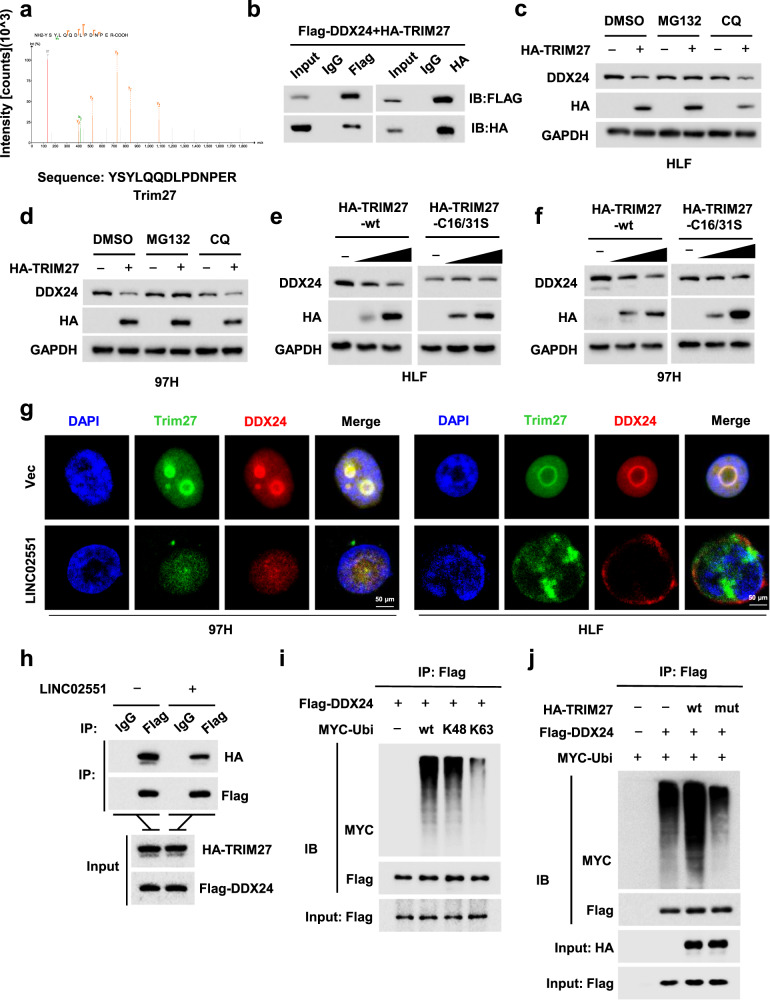
TRIM27 is a E3 ligase that mediates the ubiquitination-induced degradation of DDX24 in HCC. **a** Representative peptide sequences of TRIM27. **b** Co-IP results showing the combination of DDX24 and TRIM27 in 293 T cells. **c**, **d** HLF and 97H cells were treated with DMSO, MG132 or CQ for 4 h and endogenous DDX24 was measured by WB. **e**, **f** DDX24 expression was measured through WB in HLF and 97H cells transfected with TRIM27-wt or TRIM27-mut. **g** IF results of TRIM27 and DDX24 with or without *LINC02551* overexpression. **h** Co-IP results of TRIM27 and DDX24 with or without *LINC02551* overexpression. **i** IP results showing the combination of DDX24 and ubiquitin (wt, K48 and K63). **j** IP results showing the combination of DDX24 and ubiquitin when in cells transfected with TRIM27-wt or TRIM27-mut.

To further characterize the specific molecule that mediates DDX24 degradation, immunoprecipitation (IP) followed by MS was performed to identify potential DDX24 interaction proteins (Table S2). TRIM27 appeared to be the top hit in the MS results (Fig. 5a). The combination of DDX24 and TRIM27 was then verified in 293 T cells (Fig. 5b). MG132 treatment compensated for the downregulation of DDX24 caused by TRIM27 (Fig. 5c, d). Since TRIM27 is a E3 ubiquitin-protein ligase, an inactive TRIM27-mut was utilized to determine whether TRIM27-mediated DDX24 downregulation is ubiquitin dependent. When the TRIM27-mut was expressed in a dose gradient, the DDX24 protein level was not affected (Fig. 5e, f). IF coupled with IP showed that *LINC02551* attenuated the interaction between TRIM27 and DDX24 (Fig. 5g, h). In vitro ubiquitination assays with Ub bearing mutations on all but one lysine residue indicated that lysine 48 was necessary for TRIM27-stimulated polyubiquitination of DDX24 (Fig. 5i). The ubiquitin enrichment on DDX24 was decreased in cells when transfected with TRIM27-mut (Fig. 5j).

These data revealed that TRIM27 was the E3 ligase that mediated the ubiquitination leading to degradation of DDX24 and that *LINC02551* blocked this process.

### DDX24 is a mediator of *LINC02551* that promotes HCC progression

**Fig. 6:**
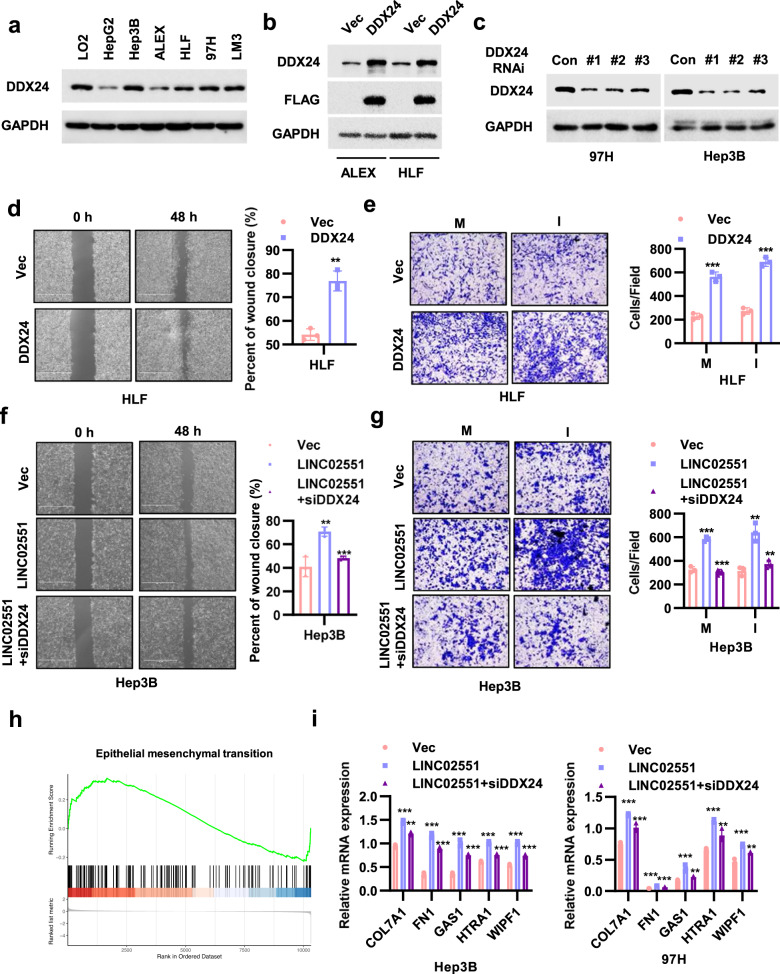
*LINC02551* exerts its effects by stabilizing DDX24 expression. **a** DDX24 protein levels were measured in HCC cell lines. **b** DDX24 expression in HLF and ALEX cells overexpressing *LINC02551*. **c** DDX24 expression in Hep3B and 97H cells transfected with shRNA-induced *LINC02551* knockdown. **d** The wound healing assay of HLF cells with DDX24 overexpression. **e** The migration and invasion results of HLF cells with DDX24 overexpression (M: migration; I: invasion). **f** The wound healing assay of Hep3B cells with *LINC02551* overexpression with or without siDDX24. **g** The migration and invasion of the cells described in f. **h** Gene set enrichment analysis showed that epithelial mesenchymal transition-related genes identified by RNA-seq were significantly enriched in HLF cells with DDX24 overexpression compared with control HLF cells. **i** The top five hit genes in EMT were selected to verify their expression in Hep3B and 97H cells with *LINC02551* overexpression with or without siDDX24. Statistical analysis was performed using Student’s *t*-tests. Data are mean ± SEM. *P < 0.05, **P < 0.001, ***P < 0.0001, ns, *P* > 0.05.

To explore the function of DDX24 in HCC, we measured its expression in different cell lines (Fig. 6a) and constructed stable overexpression and knockdown cell lines (Fig. 6b, c). In ALEX and HLF cells which express low levels of DDX24, forced overexpression of DDX24 led to increased cell motility (Fig. 6d and Fig. S4a), metastasis ability (Fig. 6e and Fig. S4b), and cell proliferation rate (Fig. S4c). To validate that the effects of *LINC02551* are partially dependent on DDX24, shRNA-mediated knockdown of DDX24 was performed with 97H and Hep3B cells. In *LINC02551* overexpressing cells, the knockdown of DDX24 partially decreased cell motility (Fig. 6f and Fig. S4d), metastasis ability (Fig. 6g and Fig. S4e), and cell proliferation rate (Fig. S4f). We next evaluated whether DDX24 exerts an effect on downstream signaling in metastasis or proliferation pathways. Microarray gene expression profiling was performed with DDX24-expressing or control 97H cells. A Kyoto Encyclopedia of Genes and Genomes (KEGG) analysis indicated that genes regulated by DDX24 overexpression were more enriched in cell adhesion molecule pathway (Fig. S2a). Gene set enrichment analysis based on our own sequencing data and TCGA data indicated that genes induced by the epithelial mesenchymal transition (EMT) were enriched to a greater degree in DDX24-overexpressing cells than in control cells (Fig. 6j and Fig. S2b). To confirm this result, a set of genes regulated by the EMT was selected. Genes upregulated by the EMT were increased in the DDX24-expressing group. Among these upregulated genes, the top five most highly upregulated were selected for use in further experiments. In Hep3B and 97H cells overexpressing *LINC02551*, the knockdown of DDX24 inhibited the upregulation that had been induced by *LINC02551* overexpression in these five genes (Fig. 6k).

These results indicated that *LINC02551* exerted its effects partially by stabilizing DDX24 expression.

### Clinical relevance of the *LINC02551*/DDX24 axis in HCC

**Fig. 7:**
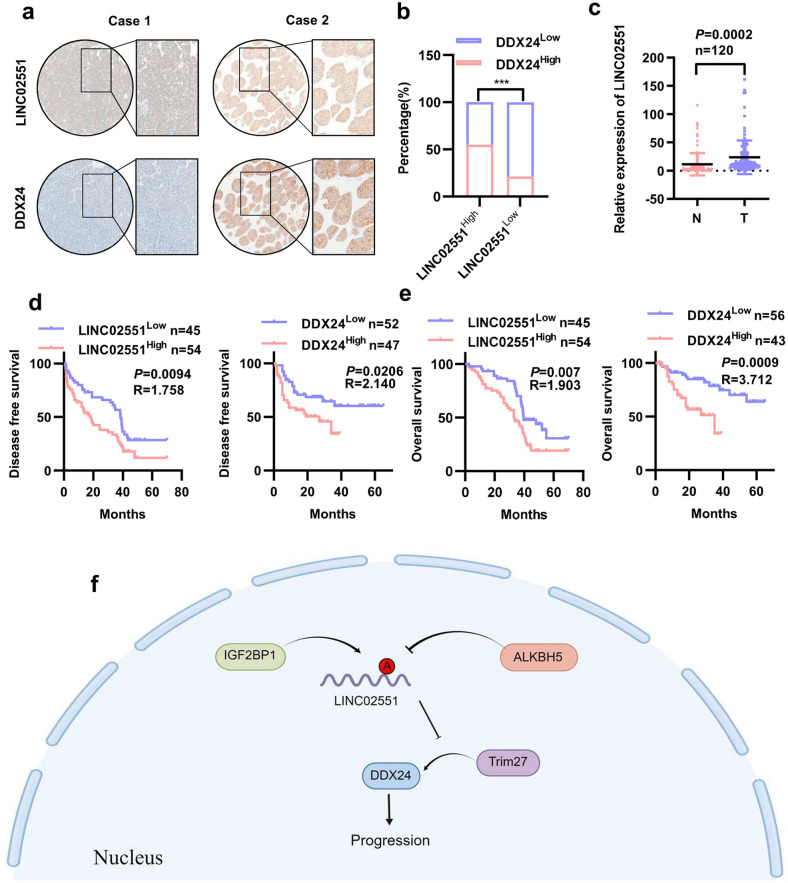
*LINC02551* is positively correlated with DDX24 expression in HCC patient samples. **a** Representative IHC staining results in paraffin-embedded HCC samples from Tongji Hospital. **b** Statistical results indicated that *LINC02551* expression was positively correlated with DDX24. *P* values were calculated by Pearson’s chi-square test. **c** Statistical analysis of the qRT-PCR results showing *LINC02551* expression in cohort 2 (*n* = 120). **d** The disease free survival prognosis of patients determined on the basis of *LINC02551* and DDX24 expression in cohort 1 (*n* = 99). **e** The overall survival prognosis of patients determined on the basis of *LINC02551* and DDX24 expression in cohort 1 (*n* = 99). *P* values represent log-rank testing of difference in cumulative survival. **f** Schematic illustration showing the roles played by *LINC02551* in HCC cells.

An IHC analysis indicated that the expression of *LINC02551* was positively correlated with the expression of DDX24 in a paraffin-embedded HCC sample array obtained from Tongji Hospital (*n* = 99) (Fig. 7a, b). Through qRT-PCR analysis, we found that *LINC02551* levels were upregulated in HCC tumor tissues in another patient cohort (*n* = 120) (Fig. 7c). When combined with the specific information on HCC patients in the latter cohort, high *LINC02551* levels were associated with a higher number of tumors (*P* = 0.039), incomplete tumor encapsulation (*P* = 0.027) and advanced BCLC stage (*P* = 0.021) (Table S3). A Kaplan-Meier analysis showed that patients with high *LINC02551* levels and high DDX24 levels were associated with a poor overall survival rate and shorter disease-free survival (Fig. 7d, e).

Ultimately, our research revealed that *LINC02551* and DDX24 are potential biomarkers for the prognostic prediction of HCC because both of them indicate a poor prognosis.

## Discussion

In the present study, we identified a lncRNA downregulated by ALKBH5 in an m^6^A-dependent manner in HCC, and this regulatory effect was mediated by IGF2BP1 recognition of m6A. *LINC02551* interacted with DDX24 in the nucleus, decreased TRIM27-induced ubiquitination-related degradation, thereby stabilizing its protein level, and ultimately promoting the EMT process.

RNA m^6^A modification affects tumor progression through various mechanisms, and “writers”, “erasers”, and “readers” play vital roles in this process [15, 29, 30]. The effect of METTL3, an important “writer”, has been extensively studied in gastrointestinal tumors [31–33]. However, the effect of ALKBH5, an “eraser”, remains poorly understood. To elucidate the mechanism of ALKBH5-induced dysregulation of lncRNAs, we performed RNA-seq to screen lncRNAs that were dysregulated by ALKBH5 overexpression. *LINC02551* appeared to be the most affected lncRNA and became our research target. An inactive mutant of ALKBH5 was used to verify that the regulatory effect was m^6^A dependent. When the enrichment of the m^6^A modification in *LINC02551* increased due to the downregulation of ALKBH5, IGF2BP1, a “reader”, recognized m6A and stabilized the degradation of the modified lncRNA. Whether other “readers” participate in this process remains to be further explored.

The function of lncRNAs depends on their subcellular location. When located in the cytoplasm, lncRNAs can act as decoys for miRNAs or combine with ribosomes and be translated into micropeptides [34, 35]. When in the nucleus, lncRNAs can combine with proteins and form functional RNA-protein complexes [36]. After determining the subcellular location of *LINC02551*, we found that nearly one-half of the *LINC02551* content was located in the nucleus (data not shown). RNA pull-down followed by MS indicated that many proteins interacted with *LINC02551*. DDX24, a DEAD box protein, appeared to be the top hit as a *LINC02551* interaction partners. Previous research demonstrated that DDX24 was a putative RNA helicase and played an inhibitory role in p53 transcriptional activity and cell proliferation arrest and senescence in breast cancer cells [37]. Emerging evidence has linked DEAD box proteins to cancer development and progression [38]. In our study, *LINC02551* decreased the ubiquitination-induced degradation of DDX24, which was dependent on the recognition of TRIM27, a member of the E3 ligase family [39]. Overexpression of DDX24 influenced a set of cell adhesion molecules and was closely related to the EMT (epithelial-mesenchymal transition), which was in line with the results of our in vitro experiments. Moreover, knockdown of DDX24 partially impeded the effects caused by *LINC02551* overexpression, suggesting that *LINC02551* promoted the progression of HCC by stabilizing DDX24 expression. However, this axis explained only the prometastatic roles of *LINC02551*, and whether the mechanism of the *LINC02551* proliferation effect in HCC is in line with that in breast cancer is unclear and remains to be further studied.

In summary, we found that ALKBH5-downregulated lncRNA *LINC02551*, which was dependent on the demethyltransferase function of ALKBH5. When the m^6^A on *LINC02551* was enriched, IGF2BP1 was recruited and recognized the m^6^A modification, leading to reduced degradation of *LINC02551*. In the nucleus, *LINC02551* bound to DDX24 and decreased its TRIM27-induced ubiquitination-related degradation. Stabilized DDX24 promoted the EMT in HCC. Our study highlights the fact that *LINC02551* is a newly discovered regulator in HCC and might serve as a potential prognostic biomarker for HCC (Fig. 7f).

## Supplementary information

Supplementary figure legends

Supplementary materials and methods

Supplementary table 1

Supplementary table 2

Supplementary table 3

Supplementary figure 1

Supplementary figure 2

Supplementary figure 3

Supplementary figure 4

Supplementary figure 5

WB (full length)

A reproducibility checklist

## Data Availability

All data generated or analyzed during this study are included either in this article or in the supplementary Materials and Methods, Tables, Figures, and Figure Legends files.
