# Supplementary material for: ALKBH5-mediated m6A modification of lincRNA LINC02551 enhances the stability of DDX24 to promote hepatocellular carcinoma growth and metastasis
Source: Cell Death Dis. 2022 Nov 5;13(11):926. doi: 10.1038/s41419-022-05386-4 (PMC9637195; doi:10.1038/s41419-022-05386-4)
Supplement: Supplementary file 1 — Supplementary figure legends [file 41419_2022_5386_MOESM1_ESM.docx]

**Fig. S1 *LINC02551* was selected as the research target. a** Clustering of differential lncRNA profiles between 97H vector cells and 97H ALKBH5 overexpressing cells. **b-c** Results from qRT-PCR validating the expression levels of 10 lncRNAs in 97H and HLF cells overexpressing ALKBH5. **d-e** qRT-PCR results of relative *LINC02551* expression in HLF cells transfected with ALKBH5 wild type (wt) or an ALKBH5 mutant (H204A) in dose gradients. **f** The verification of the ALKBH5-wt and ALKBH5-H204A transfection in 97H and HLF cells by WB. **g** Representative IHC staining of IGF2BP1 in paraffin-embedded HCC samples from Tongji Hospital (left panel). The significance of *LINC02551* and IGF2BP1 expression is indicated in the right panel. And this analysis was performed using F-test. The remaining statistical analysis was performed using Student’s t-tests. Data are mean ± SEM. **P* < 0.05, ***P* < 0.001, ****P* < 0.0001.

**Fig. S2 *LINC02551* promotes HCC progression *in vitro*. a** The migration and invasion results of Hep3B cells with *LINC02551* knocked down (M: migration; I: invasion). **b** The migration and invasion results with HLF cells overexpressing *LINC02551*. **c** The wound healing assay with Hep3B cells in which *LINC02551* was knocked down. **d** The wound healing assay with HLF cells overexpressing *LINC02551*. **e** The CCK-8 results for the aforementioned cell lines in a. **f** The CCK8 results of the cell lines described in b. Statistical analysis was performed using Student’s t-tests. Data are mean ± SEM. **P* < 0.05, ***P* < 0.001, ****P* < 0.0001.

**Fig. S3 The analysis of the combination between DDX24 and *LINC02551*. a** According to the IF and ISH confocal results, the fluorescence intensities in 97H and HLF cells were quantified

**Fig. S4 *LINC02551* promotes HCC progression through DDX24. a** The wound healing assay of ALEX cells with DDX24 overexpression. **b** The migration and invasion results of ALEX cells with DDX24 overexpression. **c** The CCK-8 results for the aforementioned cell lines in a. **d** The wound healing assay of 97H cells with *LINC02551* overexpression with or without siDDX24. **e** The migration and invasion of the cells described in d. **f** The CCK-8 results for the aforementioned cell lines in d. Statistical analysis was performed using Student’s t-tests. Data are mean ± SEM. **P* < 0.05, ***P* < 0.001, ****P* < 0.0001.

**Fig. S5 Cells signaling was affected by DDX24 overexpression. a** After RNA-seq, KEGG enrichment analysis of HLF DDX24 overexpressing cells was performed. **b** Enrichment plots showing the differences in the epithelial mesenchymal transition (EMT) between patients with high DDX24 expression and those with low DDX24 expression, as obtained from TCGA database. **c** WB analysis of EMT markers in ALEX and HLF cells with DDX24 overexpression.
