# Supplementary material for: ALKBH5-mediated m6A modification of lincRNA LINC02551 enhances the stability of DDX24 to promote hepatocellular carcinoma growth and metastasis
Source: Cell Death Dis. 2022 Nov 5;13(11):926. doi: 10.1038/s41419-022-05386-4 (PMC9637195; doi:10.1038/s41419-022-05386-4)
Supplement: Supplementary file 2 — Supplementary materials and methods [file 41419_2022_5386_MOESM2_ESM.docx]

**Cell lines and cell culture**

LM3 and 97H were obtained from the Liver Cancer Institute of Fudan University. The LO2, HepG2, Hep3B, Huh7, HLF and HEK-293T cells were purchased from the China Center for Type Culture Collection (CCTCC, Wuhan, China). These cell lines were cultured in Dulbecco’s modified Eagle’s medium (Invitrogen) supplemented with 10% fetal bovine serum (Gibco, USA) and incubated in 5% CO2 at 37 °C.

**RNA extraction and quantitative real-time PCR (qRT-PCR)**

RNA was extracted using TRIzol reagent (TaKaRa, Japan). Reverse transcription of mRNAs and LINCRNAs was performed in accordance with the manual provided by the manufacturer of a HiScriptII Q RT SuperMix for qPCR (Vazyme, China). qRT-PCR was conducted with ChamQ Universal SYBR qPCR Master Mix (Vazyme). The primers are listed in as follows:

GAPDH-F: 5’-GACAAGCTTCCCGTTCTCAG-3’

GAPDH-R: 5’-GAGTCAACGGATTTGGTCGT-3’;

LINC02551-F: 5’-GTGCTAGTCTCTGGAGCCAC-3’

LINC02551-R: 5’-CGGCCACATTTGCACCATTT-3’;

RNF207-AS1-F: 5’- CCTCAGCGCTGGGTTAAAAT-3’

RNF207-AS1-R: 5’- GCTCTCAGGACACTCAGGAG-3’;

SERTAD4-AS1-F: 5’- GACGCCTATTCCCTGCTTCT-3’

SERTAD4-AS1-R: 5’- CTCTCGCTTCCCTTCCTCC-3’;

MIR201HG-2-F: 5’- GTTCCTGTTGCCAAGCTGAA-3’

MIR201HG-2-R: 5’- GGCAGAAACACACAGAAGCA-3’;

CDIPTOSP-F: 5’- CTCCCCATCTCCCATTCTCC-3’

CDIPTOSP-R: 5’- GCAGATGGGTGGAAACCTTG-3’;

LINC01133-F: 5’- ATGGGAATGGTTGGGAGGAG-3’

LINC01133-R: 5’- ACTCCTGGGCTCAAGGAATC-3’;

ADPGK-AS1-F: 5’- CTGACCTTGGCTTTGTGTCC-3’

ADPGK-AS1-R: 5’- CAGGTAGGCTGGGTAGGATG-3’;

ITFG1-AS1-F: 5’- GCCATTTCAGACTCAGAGCAG-3’

ITFG1-AS1-R: 5’- GTTTAGGTCGGAGCGCAAAT-3’;

LINC00707-F: 5’- GCTGGACATTTTCTTGCGGA-3’

LINC00707-R: 5’- AGGTCATGGTCTTGGGAGTG-3’;

RGMB-AS1-F: 5’- AAATCTGAATGTCACCCGCG-3’

RGMB-AS1-R: 5’- ACGTTGAAGTTTGCCCACTC-3’;

DDX24-F: 5’-AGATGTGTCAGCTTGGAAGGA-3’

DDX24-R: 5’-GGTGCAGAGAAGCCTAGAAAG-3’;

COL7A1-F: 5’-TTACGCCGCTGACATTGTGTT-3’

COL7A1-R: 5’-ACCAGCCCTTCGAGAAAGC-3’;

WIPF1-F: 5’- AGCCTCAGAGGAACCGAATG-3’

WIPF1-R: 5’-CGGACTTGATTGAATGGGTCTTG-3’;

HTRA1-F: 5’-TCCCAACAGTTTGCGCCATAA-3’

HTRA1-R: 5’-CCGGCACCTCTCGTTTAGAAA-3’;

GAS1-F: 5’-ATGCCGCACCGTCATTGAG-3’

GAS1-R: 5’-TCATCGTAGTAGTCGTCCAGG-3’;

FN1-F: 5’-AGGAAGCCGAGGTTTTAACTG-3’

FN1-R: 5’-AGGACGCTCATAAGTGTCACC-3’.

**RNA interference**

LINC02551 short hairpin RNAs (sh_001: 5’-CCGGGAAAGAAGAAAGACGCACAGCTGTGCGTCTTTCTTCTTTCTTTTTG-3’, sh_002: 5’-CCGGGTTCAACGGAAATTCACAATTGTGAATTTCCGTTGAACTTTTTG-3’, sh_003: 5’-CCGGTGCCTTGAATAAAGACGTATACGTCTTTATTCAAGGCATTTTTG-3’), DDX24 small interfering RNAs (si_001: 5’- CGCTCAAGAAAGATGAGGATA-3’, si_002: 5’-TTTCTGTTCTCTGGCTATTTG-3’, si_003: 5’- CGCTCAAGAAAGATGAGGATA-3’), IGF2BP1 small interfering RNAs (si_001: 5’-GGCTCAGTATGGTACAGTA-3’, si_002: 5’-TGAAGATCCTGGCCCATAA-3’, si_003: 5’-GAAGGACGGAACCTGAAGA-3’), and ALKBH5 small interfering RNAs (si_001: 5’- GATCGCCTGTCAGGAAACA-3’, si_002: 5’-GTCCTTCTTTAGCGACTCT-3’, si_003: 5’- GCTGCAAGTTCCAGTTCAA-3’) were purchased from Ribo Bio Co., Ltd. (Guangzhou, China). Transfections with small hairpin RNAs were performed with Lipofectamine 3000 (Life Technologies).

**Cell migration and invasion assays**

For cell migration and invasion assays, Transwell chambers (8 μm pore size, Corning, NY, USA) were placed in a 24-well culture plate with 600 μl of DMEM supplemented with 10% FBS added to the lower chambers. Cells in 100 μl of serum-free DMEM were cultured in the upper chamber. After incubation for 24 h at 37 °C, the migrated cells on the lower surface of the membrane were fixed with 4% paraformaldehyde and stained with 0.1% crystal violet. Precoated Matrigel (BD Biosciences, CA, USA) were precoated on the upper chamber for the invasion assay.

**Cell Counting Kit-8 (CCK-8) assay**

Cell Counting Kit-8 (Dojindo, Japan) was used to evaluate cell viability. Briefly, 1000 cells were cultured in 96-well plates. At the indicated time, cells were incubated with 10% CCK-8 solution for 1h at 37℃. The absorbance was measured using a microplate reader (Bio-Tek Instruments, USA) at 450 nm.

**Western blotting (WB)**

Protein was extracted using RIPA buffer (Meilunbio, China) supplemented with 1% protease and 1% phosphatase inhibitor cocktail. After centrifugation at 12,000 × g for 15 min, the supernatant at a final concentration of 2 μg/μl was boiled at 100℃ for 15 min. The protein was separated on SDS-PAGE gels. After incubation with primary antibodies and HRP-conjugated secondary antibodies (Aksomics, China), protein bands were detected by the ECL Detection System (Bio–Rad, USA). The following antibodies were: anti-GAPDH (60004-1-Ig, Proteintech), anti-ALKBH5 (16837-1-AP, Proteintech), anti-IGF2BP1 (22803-1-AP, Proteintech), anti-FLAG ((F1804, Sigma), anti-HA (H6908, Sigma), anti-MYC-Tag (2276, CST, USA), anti-DDX24 (15769-1-AP, Proteintech)

**Luciferase reporter assay**

Cells were cultured in 24-wells plates and were transfected with LINC02551-wt reporter and potential m6A sites mutant LINC02551 reporter plasmids using Lipofectamine 3000 (Invitrogen, USA). The luciferase activity was performed using a Dual-Luciferase Reporter Assay System (E1910, Promega). The Renilla luciferase activity was normalized to firefly luciferase activity.

**In situ hybridization (ISH) and imunohistochemistry (IHC)**

ISH was performed to assess LINC02551 expression in formalin-fixed, paraffin-embedded samples. To analyze the expression of LINC02551 in paraffin-embedded hepatocellular carcinoma tissues, we designed three probes with 5’-digoxigenin-labeled oligonucleotides

(5’-GGAAGACCTAGGAAGCTAGCGTCCGG-3’; 5’-CAGAGAACTTGTTCTTGGCTTCTGCTACTAGAGAA-3’; and 5’-CCTCGGCCACATTTGCACCATTTCTTTTC-3’) for hybridization with LINC02551 in situ using an Enhanced Sensitive ISH Detection Kit (Boster Biology Company, Wuhan, Hubei Province, China) following the manufacturer’s instructions.. IHC staining was performed using a polymer HRP detection system (Zhongshan Goldenbridge Biotechnology, China). The primary antibodies used for IHC include anti-ALKBH5 (16837-1-AP, Proteintech, China), anti-E-Cadherin (07-697, Sigma, USA), anti-N-Cadherin (05-915, Sigma), anti-Vimentin (V6389, Sigma), anti-Snail (SAB5700796, Sigma), anti-Ki67 (ab15580, abcam, USA), anti-PCNA (MA1083, Boster), anti-DDX24 (15769-1-AP, Proteintech).

**Immunofluorescence (IF) and fluorescence in situ hybridization (FISH)**

For immunofluorescence measurements, cells were fixed in 4% paraformaldehyde, permeabilized with 0.1% Triton X-100, and blocked with 3% bovine serum albumin (BSA). The indicated cells were incubated with primary antibody against DDX24 (Proteintech) at 4 ℃ overnight. Then cells were incubated with fluorophore-conjugated secondary antibodies and nuclei were counterstained with DAPI.

For fluorescence in situ hybridization, Cy3-labeled probes specific for LINC02551 were synthesized by RiboBio (Guangzhou, China). A fluorescent in situ hybridization kit (RiboBio) was used to hybridize the probes to cells. Images were acquired with a laser scanning confocal microscope (LSM710, Carl Zeiss, Germany).

**Methylated RNA immunoprecipitation (MeRIP)**

MeRIP assay was performed using Magna MeRIP™ m6A kit (17-10499, Millipore) according to the manufacturer’s protocol. Briefly, total RNA was extracted using TRIzol. Then RNA was fragmented into ~100-nucleotide-long fragments by sonication and then, magnetic immunoprecipitation was performed with a monoclonal antibody against m6A (EMD Millipore, Temecula, CA, MABE1006). After immunoprecipitation, enriched RNA fragments were analyzed with qRT-PCR.

**MS2-FLAG-RNA-immunoprecipitation**

The pcDNA3.1 plasmid was fused with the 6×MS2-binding sequence via homologous to construct a pcDNA3.1-stem loop plasmid. The *LINC02551* sequence and the indicated truncated *LINC02551* sequence were inserted into the pcDNA3.1-stem loop between EcoRI and XbaI. Then, 10 μg of MS2-FLAG and pcDNA-st-LINC02551 plasmids was cotransfected into the corresponding HEK293 cells and the cells were extracted 48 h later. Subsequently, cell lysis buffer was precleared with 50 μl of protein G-agarose (Santa Cruz, USA) at 4 °C for 2 h. The supernatant was then incubated with Flag antibodies with gentle shaking at 4 °C, overnight. This was followed by the addition of 50 μl of protein A/G-agarose for another 2 h. Finally, the beads were washed and resuspended in 50 μl of 2×SDS-PAGE loading buffer and boiled for 10 min before Western blotting.

**Coimmunoprecipitation (Co-IP)**

Cells were lysed with IP lysis buffer (50 mM Tris-HCl, 150 mM NaCl, 1% Triton X-100, 1 mM EDTA, 10% glycerol, and protease inhibitor cocktail, pH 7.4). Supernatants were collected by centrifugation (13,000 g, 15 min, 4 °C) and incubated with the indicated antibodies (1µg/ml) overnight at 4 °C, followed by immunoprecipitation with 20 µl of protein G-conjugated agarose beads for 2 h at 4 °C. The precipitates were washed 4 times with IP-wash buffer (50 mM Tris-Cl, 300 mM NaCl, 1% Triton X-100, 1 mM EDTA, pH 7.4) and detected by Western blotting.

**Animal experiments**

All of the animal studies were approved by the Committee on the Ethics of Animal Experiments of the Tongji Medical College, HUST. Three mouse models were used in this study. For establishing the subcutaneous xenograft model, 1×10^6^ HCC cells were injected subcutaneously into the axillae of 4-week-old male BALB/C nude mice to generate tumor xenografts. For establishing the orthotopic xenograft tumor metastasis model, luciferase-bearing HCC cells (1×10^6^) were injected into the left lobe of livers of 4-week-old male BALB/C nude mice and the mice were sacrificed 4 weeks later. For establishing the lung metastasis model, we slowly injected HCC cells (1×10^6^ cells/100 μl) into 5-week-old male BALB/c nude mice via the tail vein. Six weeks after injection, luciferase bioluminescence was measured, and the mice were then sacrificed.

**Statistical analysis**

The data are presented as the mean ± standard deviation (SD). SPSS 21.0 or GraphPad Prism 8.0 software was used to analyze the statistical significance of the data. Student’s t test (two tails) was used to determine the statistical significance of differences between groups. Survival rates were determined using the Kaplan–Meier method with log-rank tests. Pearson χ2 and Fisher’s exact test were performed to analyze the categorical data. A *P* value＜0.05 indicated statistical significance.
