# Supplementary material for: ALKBH5-mediated m6A modification of lincRNA LINC02551 enhances the stability of DDX24 to promote hepatocellular carcinoma growth and metastasis
Source: Cell Death Dis. 2022 Nov 5;13(11):926. doi: 10.1038/s41419-022-05386-4 (PMC9637195; doi:10.1038/s41419-022-05386-4)
Supplement: Supplementary file 3 — Supplementary table 1 [file 41419_2022_5386_MOESM3_ESM.docx]

**Supplementary Table 1. Proteins binding with LINC02551 identified by IP-MS.**

| Entry name | # Unique Peptides | # AAs | MW [kDa] |
| --- | --- | --- | --- |
| **DDX24_HUMAN** | 4 | 859 | 96.3 |
| GRP75_HUMAN | 4 | 679 | 73.6 |
| NVL_HUMAN | 4 | 856 | 95 |
| EXOSX_HUMAN | 4 | 885 | 100.8 |
| NEMF_HUMAN | 4 | 1076 | 122.9 |
| NSUN2_HUMAN | 3 | 767 | 86.4 |
| SND1_HUMAN | 3 | 910 | 101.9 |
| PUM3_HUMAN | 3 | 648 | 73.5 |
| RS17_HUMAN | 3 | 135 | 15.5 |
| HNRPM_HUMAN | 3 | 730 | 77.5 |
| DDX5_HUMAN | 3 | 614 | 69.1 |
| POP1_HUMAN | 3 | 1024 | 114.6 |
| RL4_HUMAN | 3 | 427 | 47.7 |
| SRPK1_HUMAN | 3 | 655 | 74.3 |
| UBP2L_HUMAN | 3 | 1087 | 114.5 |
| HNRPU_HUMAN | 3 | 825 | 90.5 |
| RL13_HUMAN | 3 | 211 | 24.2 |
| UBAP2_HUMAN | 3 | 1119 | 117 |
| YTDC2_HUMAN | 3 | 1430 | 160.1 |
| MA7D1_HUMAN | 3 | 841 | 92.8 |
| HNRLL_HUMAN | 3 | 542 | 60 |
| SRRM1_HUMAN | 3 | 904 | 102.3 |
| IMA1_HUMAN | 3 | 529 | 57.8 |
| SRP68_HUMAN | 3 | 627 | 70.7 |
| NUFP2_HUMAN | 3 | 695 | 76.1 |
| LYRIC_HUMAN | 3 | 582 | 63.8 |
| RS7_HUMAN | 3 | 194 | 22.1 |
| GGYF2_HUMAN | 3 | 1299 | 150 |
| DHX57_HUMAN | 3 | 1386 | 155.5 |
| XRN2_HUMAN | 3 | 950 | 108.5 |
| SF3B3_HUMAN | 3 | 1217 | 135.5 |
| HS71B_HUMAN | 3 | 641 | 70 |
| GRWD1_HUMAN | 3 | 446 | 49.4 |
| PRC2C_HUMAN | 3 | 2896 | 316.7 |
| TBA8_HUMAN | 1 | 449 | 50.1 |
| ZCCHV_HUMAN | 3 | 902 | 101.4 |
| TOP2A_HUMAN | 3 | 1531 | 174.3 |
| HNRPC_HUMAN | 3 | 306 | 33.7 |
| MK67I_HUMAN | 3 | 293 | 34.2 |
| EIF3L_HUMAN | 3 | 564 | 66.7 |
| SRP72_HUMAN | 3 | 671 | 74.6 |
| IF2B1_HUMAN | 3 | 577 | 63.4 |
| HNRPQ_HUMAN | 3 | 623 | 69.6 |
| TR150_HUMAN | 3 | 955 | 108.6 |
| DHX30_HUMAN | 3 | 1194 | 133.9 |
| RBM25_HUMAN | 3 | 843 | 100.1 |
| DDX41_HUMAN | 3 | 622 | 69.8 |
| SRP54_HUMAN | 3 | 504 | 55.7 |
| PESC_HUMAN | 3 | 588 | 68 |
| LARP4_HUMAN | 3 | 724 | 80.5 |
| RL1D1_HUMAN | 3 | 490 | 54.9 |
| RL26_HUMAN | 3 | 145 | 17.2 |
| HNRPR_HUMAN | 3 | 633 | 70.9 |
| RFC1_HUMAN | 3 | 1148 | 128.2 |
