# Supplementary material for: ALKBH5-mediated m6A modification of lincRNA LINC02551 enhances the stability of DDX24 to promote hepatocellular carcinoma growth and metastasis
Source: Cell Death Dis. 2022 Nov 5;13(11):926. doi: 10.1038/s41419-022-05386-4 (PMC9637195; doi:10.1038/s41419-022-05386-4)
Supplement: Supplementary file 4 — Supplementary table 2 [file 41419_2022_5386_MOESM4_ESM.docx]

**Supplementary Table 2. Proteins binding with DDX24 identified by IP-MS.**

| **Entry name** | **#Unique peptides** | **#AAs** | **MW [kDa]** |
| --- | --- | --- | --- |
| DDX24_HUMAN | 1 | 859 | 96.332 |
| MND1_HUMAN | 1 | 165 | 18.984 |
| RPL21_HUMAN | 4 | 160 | 18.565 |
| TRIM27_HUMAN | 10 | 513 | 58.49 |
| RPL36_HUMAN | 4 | 105 | 12.254 |
| RPL32_HUMAN | 5 | 133 | 15.617 |
| STAU1_HUMAN | 10 | 494 | 54.709 |
| RPL3_HUMAN | 13 | 403 | 46.109 |
| GNL3_HUMAN | 10 | 549 | 61.993 |
| RRP1B_HUMAN | 8 | 758 | 84.428 |
| RPL7A_HUMAN | 16 | 266 | 29.996 |
| LTV1_HUMAN | 7 | 475 | 54.855 |
| C7orf50_HUMAN | 3 | 192 | 21.883 |
| RPL22L1_HUMAN | 2 | 121 | 14.478 |
| RPL10A_HUMAN | 11 | 217 | 24.831 |
| YBX3_HUMAN | 7 | 372 | 40.09 |
| RPL8_HUMAN | 6 | 257 | 28.025 |
| ZC3HAV1_HUMAN | 7 | 902 | 101.431 |
| MMTAG2_HUMAN | 5 | 263 | 29.412 |
| RPL35A_HUMAN | 4 | 110 | 12.538 |
| SSB_HUMAN | 7 | 408 | 46.837 |
| RPL15_HUMAN | 8 | 204 | 24.146 |
| DDX21_HUMAN | 12 | 783 | 87.344 |
| RPL18A_HUMAN | 6 | 141 | 16.715 |
| TOP1_HUMAN | 7 | 765 | 90.726 |
| SRP9_HUMAN | 1 | 49 | 5.769 |
| RPL23A_HUMAN | 7 | 156 | 17.695 |
| RSL1D1_HUMAN | 4 | 430 | 48.209 |
| NOP2_HUMAN | 5 | 812 | 89.302 |
| RPS23_HUMAN | 4 | 143 | 15.808 |
| NCL_HUMAN | 21 | 710 | 76.614 |
| DHX37_HUMAN | 6 | 1157 | 129.545 |
| RPL6_HUMAN | 11 | 288 | 32.728 |
| RPL27_HUMAN | 4 | 136 | 15.798 |
| RPL34_HUMAN | 3 | 117 | 13.293 |
| RPL24_HUMAN | 5 | 121 | 14.369 |
| RPL27A_HUMAN | 4 | 108 | 12.201 |
| RPL12_HUMAN | 5 | 165 | 17.819 |
| RPL26_HUMAN | 9 | 145 | 17.258 |
| RPL29_HUMAN | 2 | 159 | 17.752 |
| RPL18_HUMAN | 4 | 164 | 18.756 |
| RPL7_HUMAN | 12 | 248 | 29.226 |
| H1-10_HUMAN | 2 | 213 | 22.487 |
| LACTB_HUMAN | 2 | 547 | 60.694 |
| RPL17_HUMAN | 8 | 169 | 19.586 |
| SNU13_HUMAN | 2 | 128 | 14.174 |
| DDX31_HUMAN | 4 | 746 | 83.516 |
| MYBBP1A_HUMAN | 6 | 1328 | 148.855 |
| ASPH_HUMAN | 4 | 758 | 85.863 |
| RPL14_HUMAN | 6 | 124 | 14.558 |
