# Supplementary material for: ALKBH5-mediated m6A modification of lincRNA LINC02551 enhances the stability of DDX24 to promote hepatocellular carcinoma growth and metastasis
Source: Cell Death Dis. 2022 Nov 5;13(11):926. doi: 10.1038/s41419-022-05386-4 (PMC9637195; doi:10.1038/s41419-022-05386-4)
Supplement: Supplementary file 5 — Supplementary table 3 [file 41419_2022_5386_MOESM5_ESM.docx]

**Supplementary Table 3. Correlation between LINC02551 and clinicopathological characteristics in HCC (n=120).**

| **Clinicopathological**  **variables** | **Relative LINC02551 Expression** | | **P value** |
| --- | --- | --- | --- |
|  | **Low** | **High** |  |
| **Gender**  Male  Female | 45  8 | 58  9 | 0.799 |
| **Age**  ≤50  > 50 | 24  29 | 25  42 | 0.455 |
| **AFP (ug/L)**  ≤20  > 20 | 15  38 | 11  56 | 0.126 |
| **GGT(u/l)**  ≤54  > 54 | 16  37 | 21  46 | 1.000 |
| **ALT(ng/ml)**  ≤75  >75 | 46  7 | 58  9 | 1.000 |
| **HBV**  Negative  Positive | 10  53 | 14  67 | 0.822 |
| **HCV**  Negative  Positive | 52  1 | 66  1 | 0.868 |
| **Cirrhosis**  No  Yes | 7  46 | 12  55 | 0.616 |
| **Tumor size (cm)**  ≤5  >5 | 28  25 | 23  44 | **0.062** |
| **Tumor encapsulation**  Complete  None | 35  18 | 30  37 | **0.027** |
| **Tumor number**  Single  Multiple | 44  9 | 44  23 | **0.039** |
| **Vascular invasion**  No  Yes | 46  7 | 48  19 | 0.073 |
| **Differentiation**  I- II  III-IV | 44  9 | 53  14 | 0.646 |
| **BCLC stage**  0+A  B+C | 41  12 | 38  29 | **0.021** |
