# Supplementary figures and images for: ALKBH5-mediated m6A modification of lincRNA LINC02551 enhances the stability of DDX24 to promote hepatocellular carcinoma growth and metastasis
Source: Cell Death Dis. 2022 Nov 5;13(11):926. doi: 10.1038/s41419-022-05386-4 (PMC9637195; doi:10.1038/s41419-022-05386-4)

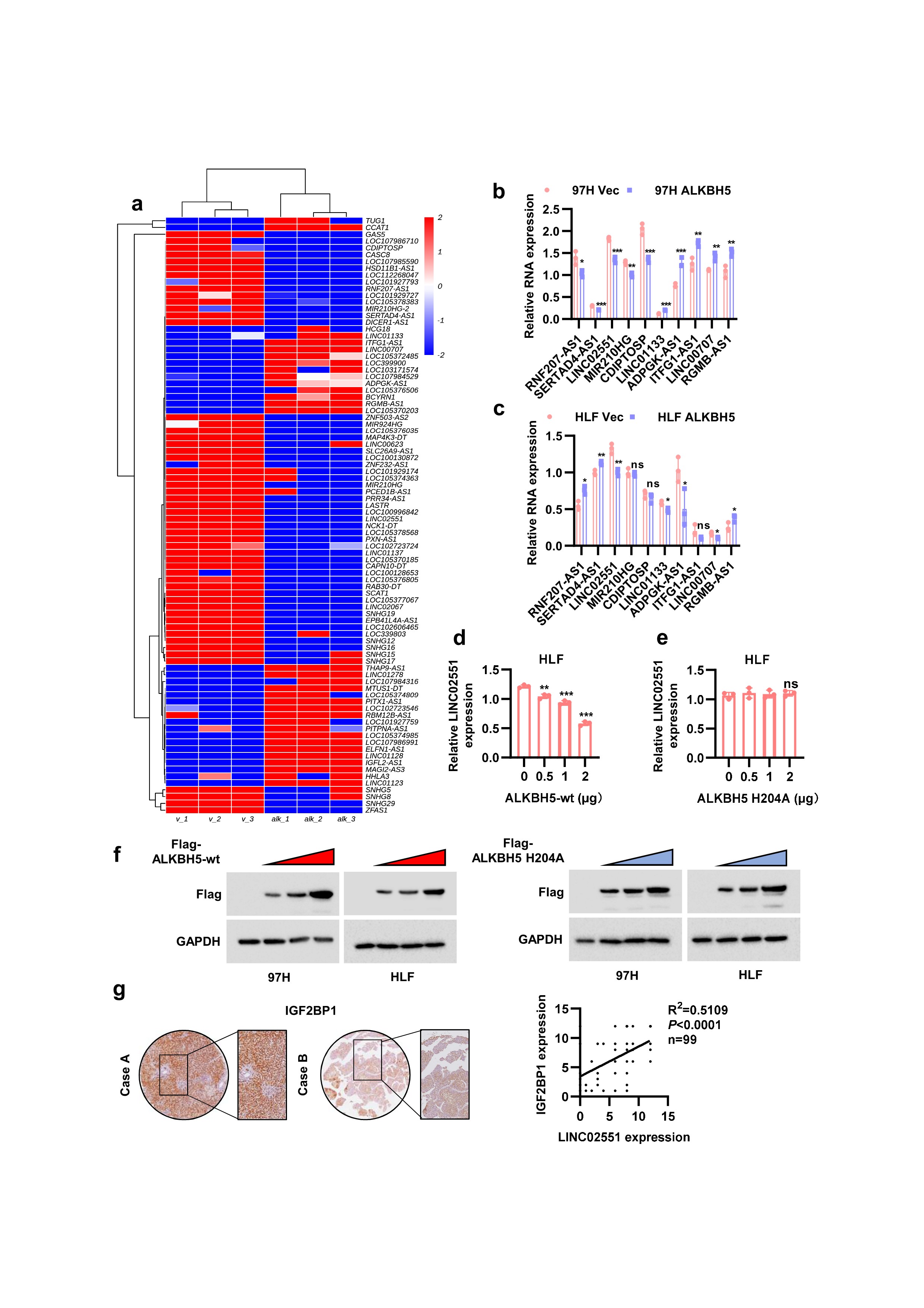

Supplement: Supplementary file 6 — Supplementary figure 1 [file 41419_2022_5386_MOESM6_ESM.jpg]

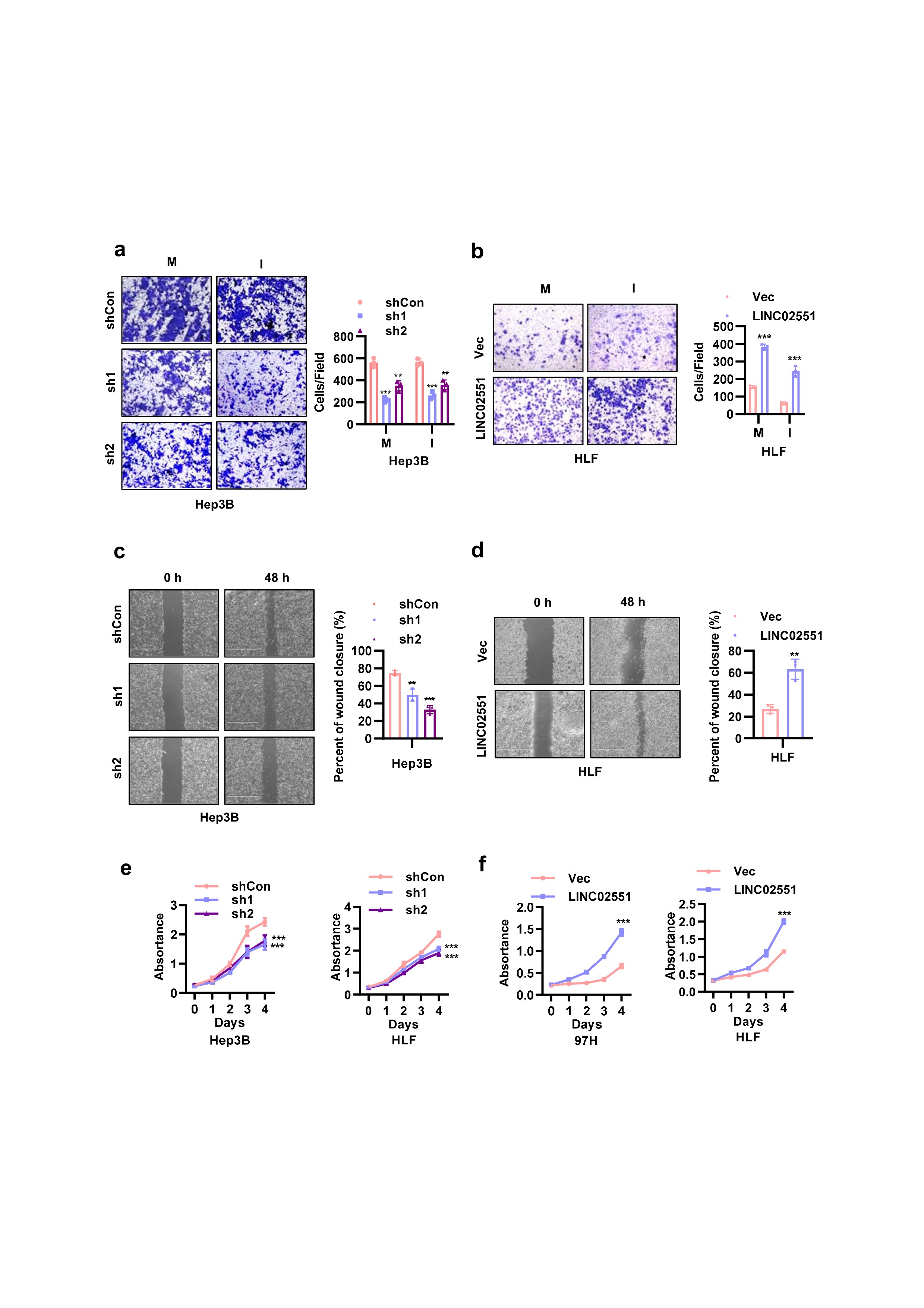

Supplement: Supplementary file 7 — Supplementary figure 2 [file 41419_2022_5386_MOESM7_ESM.jpg]

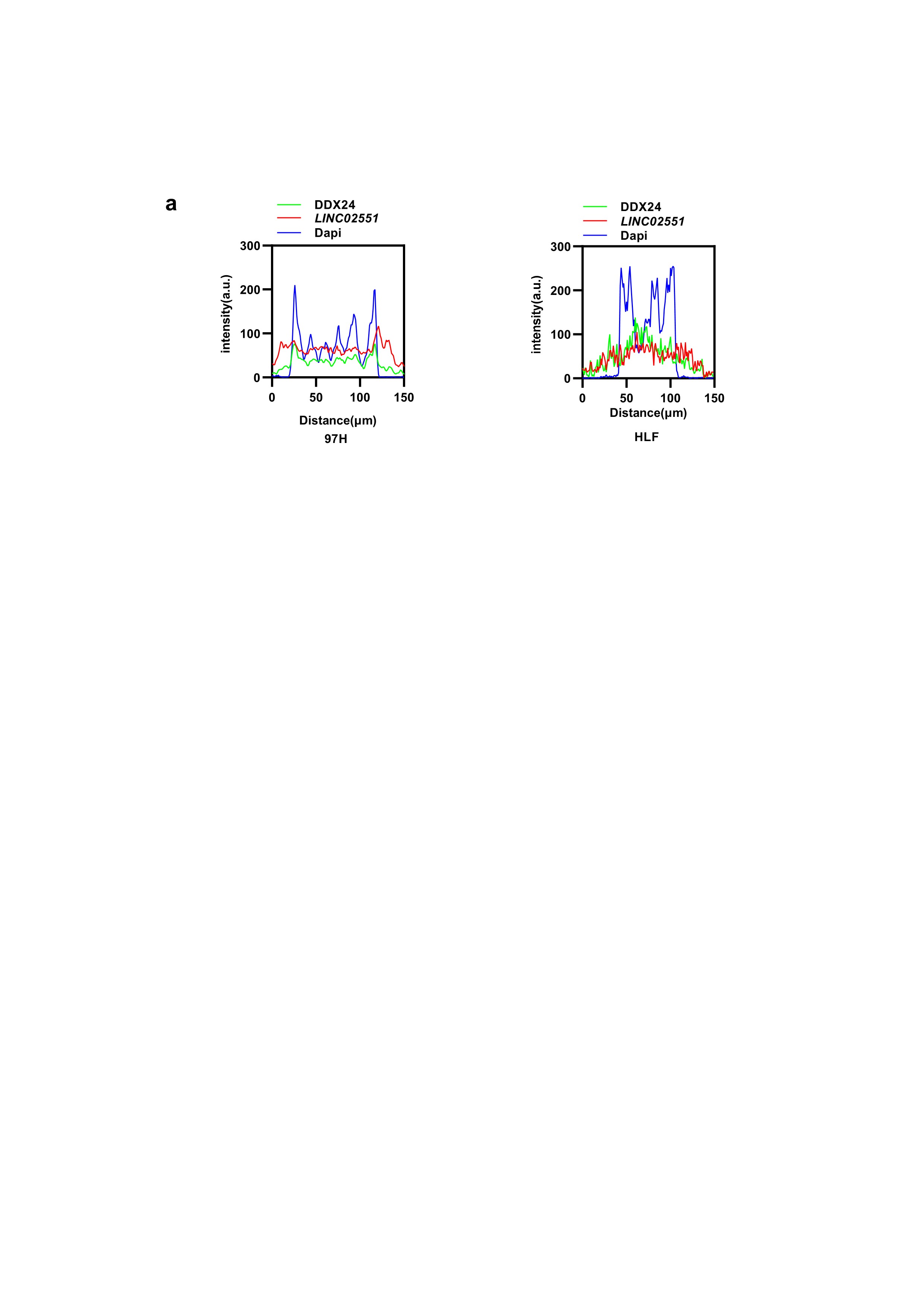

Supplement: Supplementary file 8 — Supplementary figure 3 [file 41419_2022_5386_MOESM8_ESM.jpg]

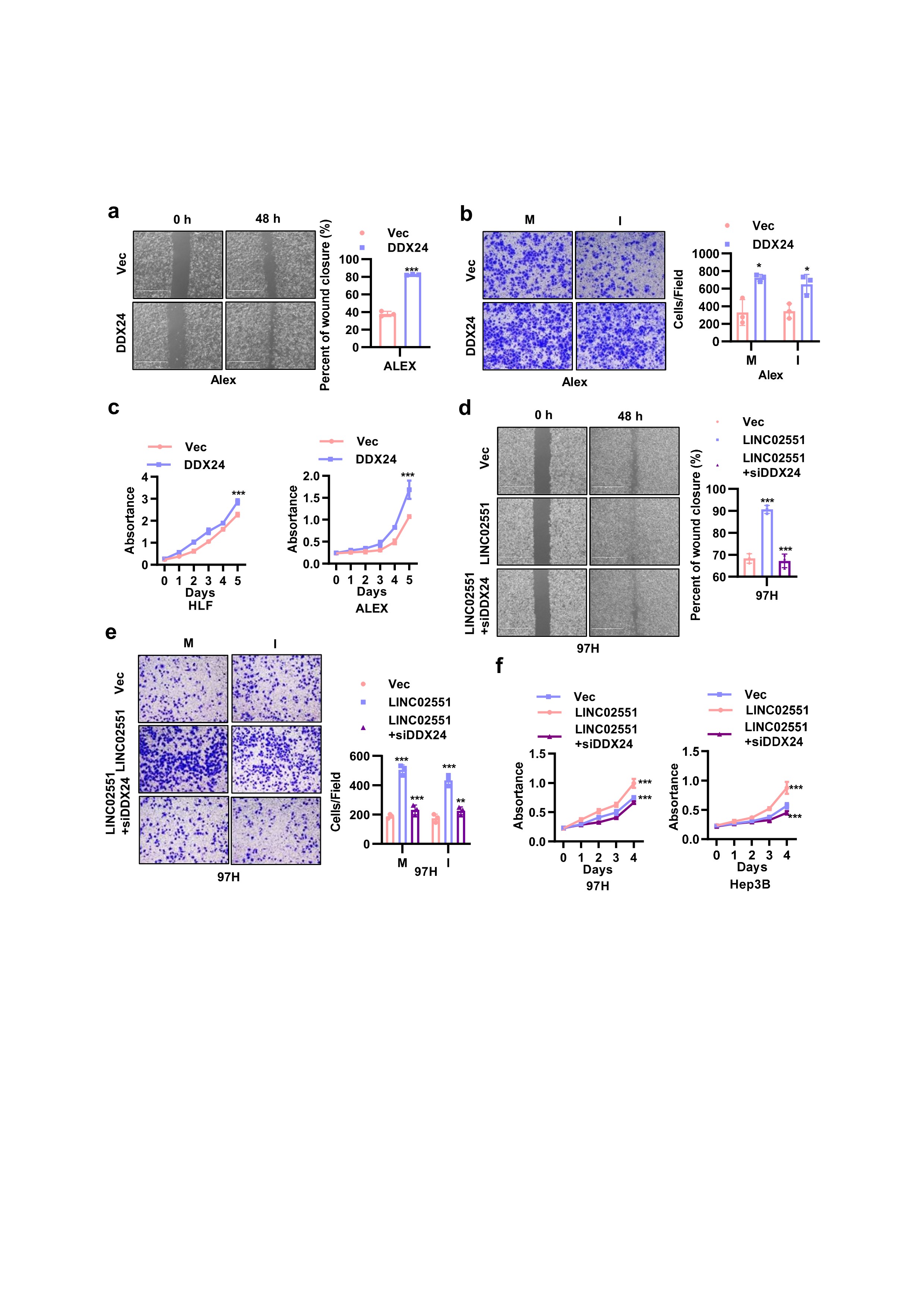

Supplement: Supplementary file 9 — Supplementary figure 4 [file 41419_2022_5386_MOESM9_ESM.jpg]

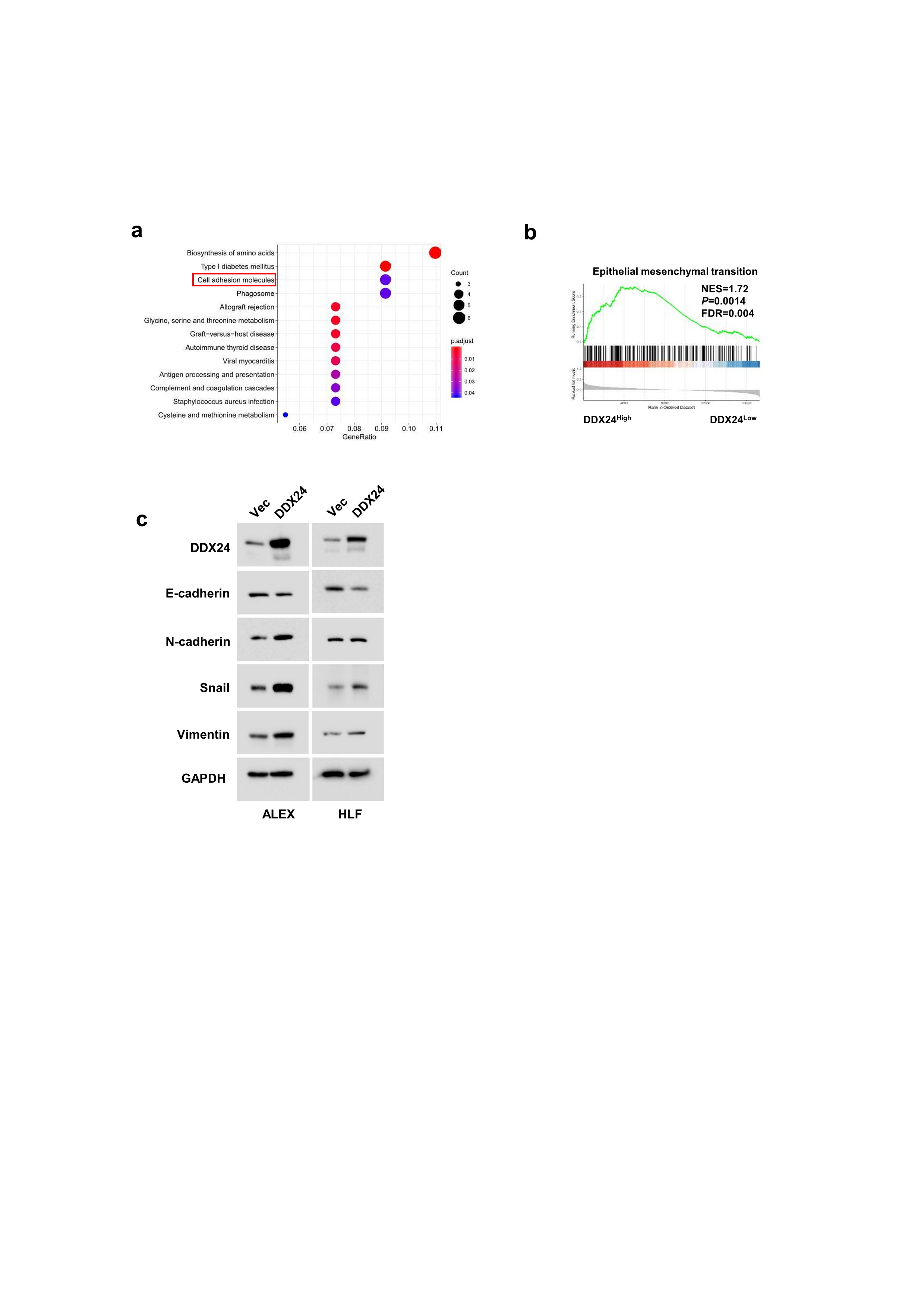

Supplement: Supplementary file 10 — Supplementary figure 5 [file 41419_2022_5386_MOESM10_ESM.jpg]

1K

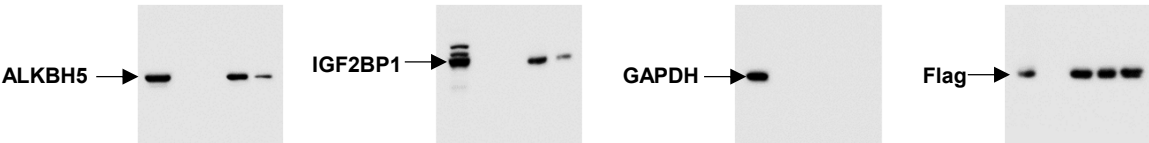

S1f

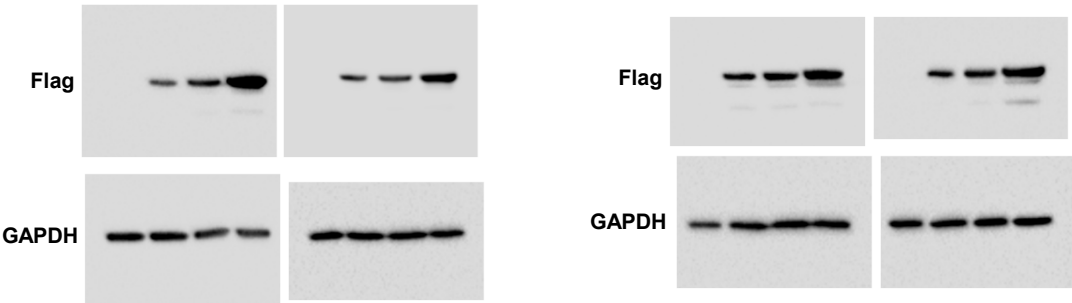

Full and uncropped western blot for Figure 4

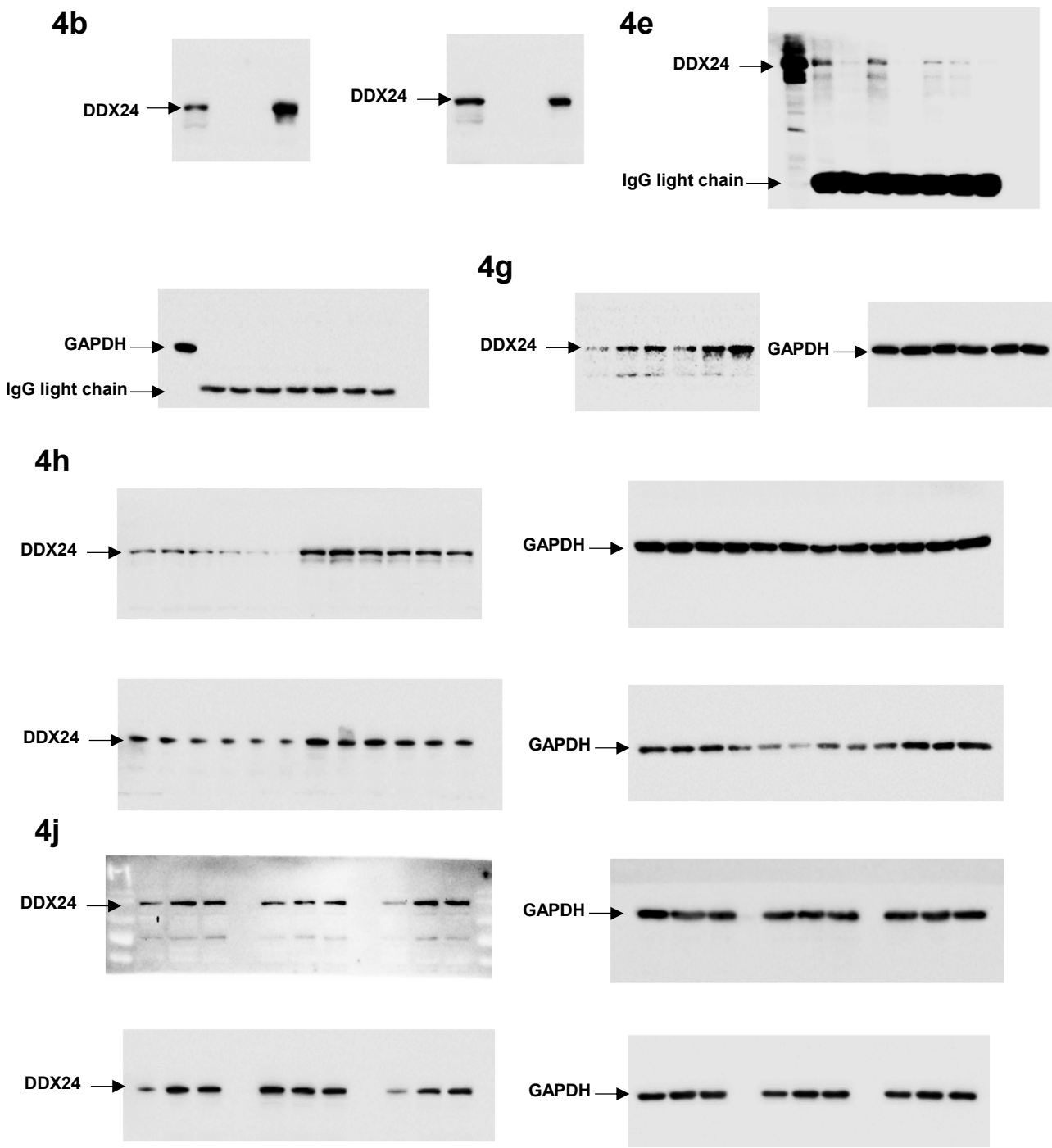

5b

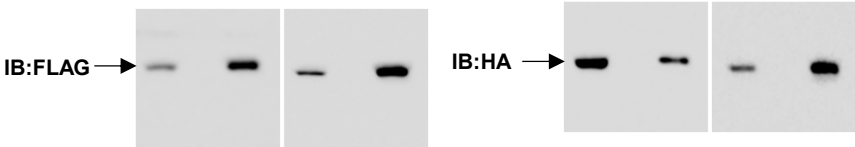

5c

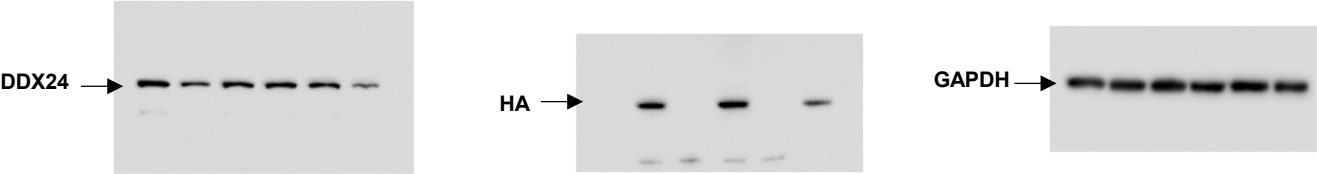

5d

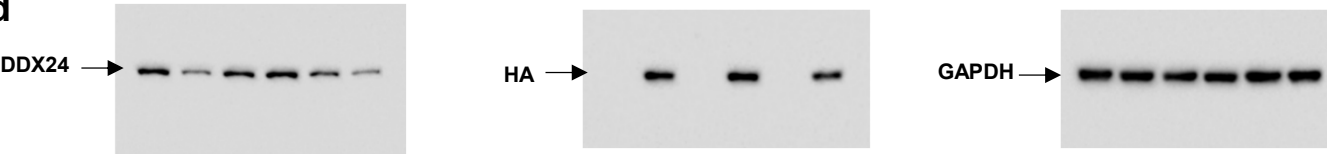

5e

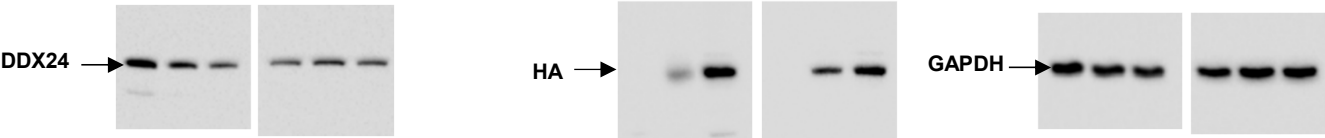

5f

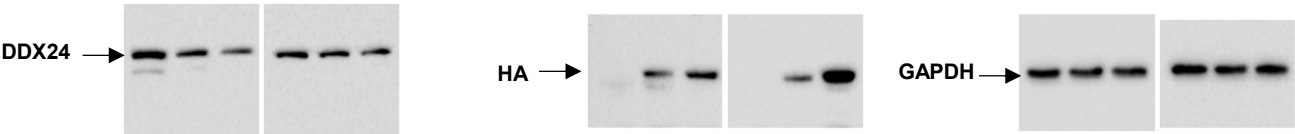

5h

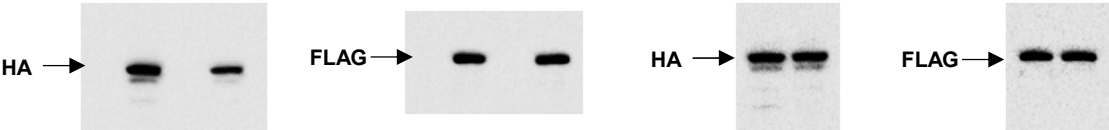

5i

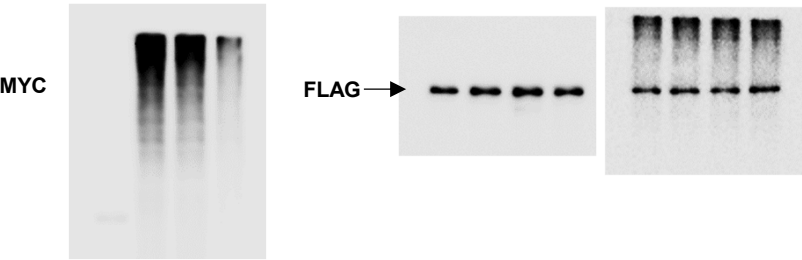

5j

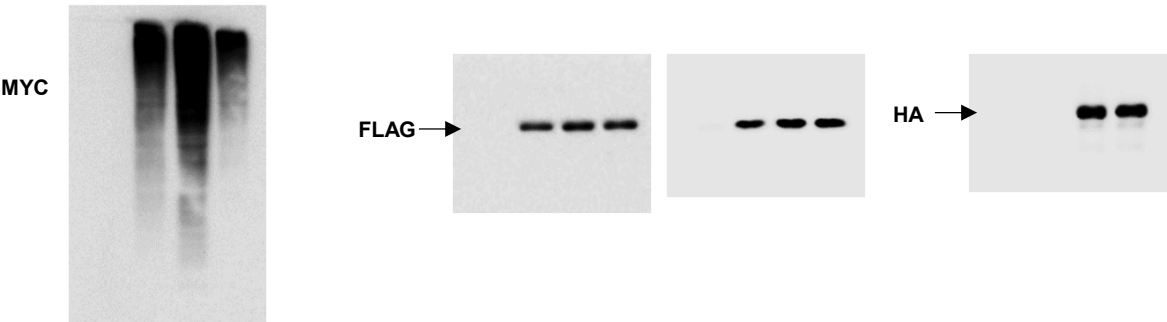

6a

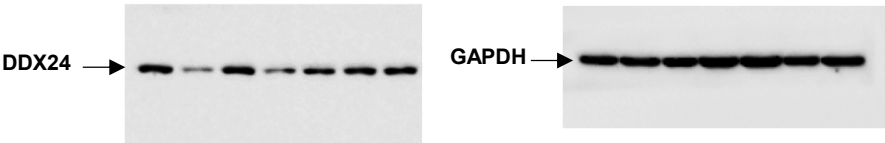

6b

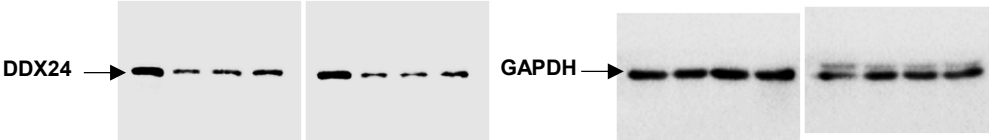

6c

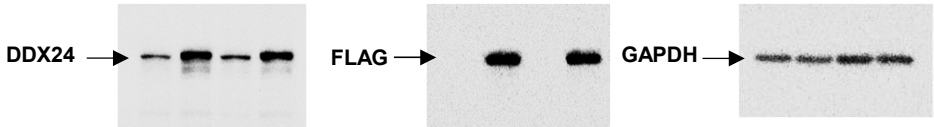

s5c

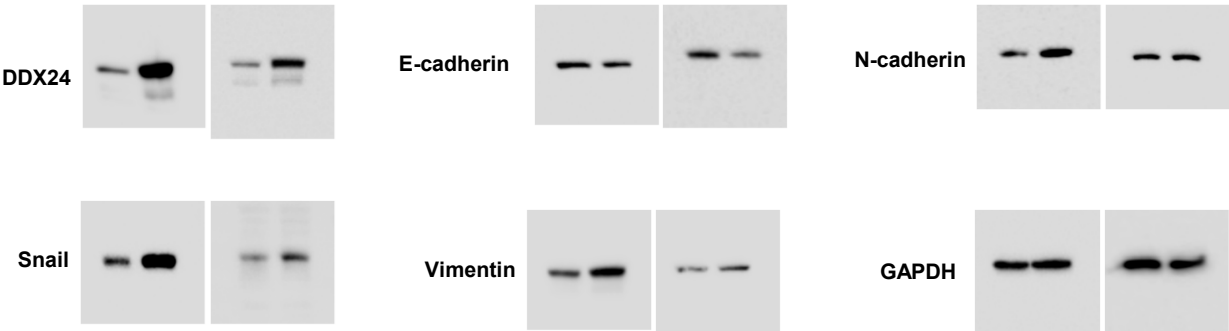

Supplement: Supplementary file 11 — WB (full length) [file 41419_2022_5386_MOESM11_ESM.pdf]
